# Structural basis of protease-activated receptor 2 activation and biased agonism

**DOI:** 10.1038/s41421-025-00851-8

**Published:** 2025-12-02

**Authors:** Xinyan Zhu, Ruixue Xia, Anqi Zhang, Changyou Guo, Zhenmei Xu, Yuanzheng He

**Affiliations:** 1 HIT Center for Life Sciences, School of Life Science and Technology, Faculty of Life Sciences and Medicine, Harbin Institute of Technology, Harbin, Heilongjiang China; 2 Frontiers Science Center for Matter Behave in Space Environment, Harbin Institute of Technology, Harbin, Heilongjiang China

**Keywords:** Cryoelectron microscopy, Hormone receptors

## Abstract

Protease-activated receptor 2 (PAR2) is a transmembrane receptor that is irreversibly activated by proteolytic cleavage of its N-terminus via extracellular proteases, resulting in the release of the tethered ligand (TL), which binds to and activates the receptor. PAR2 plays a pivotal role in the inflammatory response and pain sensation and is a promising drug target for treating arthritis, asthma, and neuronal pain. Here, we present the cryo-electron microscopy structures of active PAR2 complexed with miniG_s/q_ and miniG_13_. Combining functional assays with structural analysis, our study revealed that TL forms a parallel β-sheet with the extracellular loop 2 of PAR2 to engage the receptor. The binding of TL triggers a conformational rearrangement in the transmembrane core, releasing the inhibitory ion lock and allowing receptor activation. Furthermore, we provide structural insights into the engagement of G_q_ and G_13_ with PAR2, highlighting that a hydrophobic interaction mediated by the last methionine residue of Gα_13_ is crucial for G_13_ coupling selectivity. In combination with molecular dynamics simulations and mutagenesis, we identified the I39^TL3^/D62^N-term^ interaction at the pocket side of the receptor as a key determinant of G_13_ signaling. Disrupting this interaction significantly inhibits G_13_ signaling while preserving G_q_ activity, enabling us to design a biased peptide ligand that selectively activates G_q_ signaling. The information revealed in this study provides a framework for understanding PAR2 signaling and offers a rational basis for the design of biased PAR2 ligands.

## Introduction

The family of protease-activated receptors (PARs), which include 4 members (PAR1–4), are G protein-coupled receptors (GPCRs) that are irreversibly activated by proteases^1–4^. Proteolytic cleavage of PAR exposes a truncated N-terminus, which acts as a “tethered ligand (TL)” that binds to and subsequently activates the receptor^4^ via a similar mechanism by which adhesion GPCRs (aGPCRs) are activated by the “stalk peptide”^5^. While PAR1 is widely expressed throughout the body and generally activates G_12/13_, G_i_, and G_q_ signaling pathways, PAR2 is widely expressed in endothelial, epithelial, immune, and smooth muscle cells. PAR2 primarily signals through G_q_ and G_12/13_^4,6^. PAR2 has been implicated in various physiological and pathophysiological processes, including inflammatory responses, pain sensation, cellular permeability, contractility, and cancer development^4^. Given its diverse roles, PAR2 is considered an attractive target for therapeutic interventions in conditions such as rheumatoid arthritis, asthma, chronic pain, inflammatory bowel diseases, and neurological disorders^4,7^.

PAR1 is activated mainly by thrombin but can also be activated by matrix metalloprotease 1 (MMP1), elastase, and activated protein C (APC). PAR2 can be activated by various extracellular proteases, including trypsin, tryptase, cathepsin S, granzyme A, factor VIIa, matriptase, and neutrophil elastase^4,8^. The most common activation mechanism for PAR2 involves proteolysis at the “canonical” R36↓S37 site by trypsin and tryptase. Additionally, cathepsin S (CS) activates PAR2 through E56↓T57 cleavage, whereas neutrophil elastase (NE) triggers receptor activation through cleavage between S67↓V68^4^. Notably, the NE cleavage site (S67↓V68) is adjacent to TM1, which leads to PAR2 activation through a non-tethered peptide ligand mechanism^9^. Interestingly, different cleavage sites on PAR2 are associated with distinct signaling outcomes. Canonical cleavage at R36↓S37 activates all known PAR2 signaling pathways, including those mediated by G_q/11_, G_12/13_, and G_i_^10,11^. In contrast, CS cleavage at E56↓T57 induces cAMP accumulation without triggering Ca^2+^ release, whereas NE cleavage at S67↓V68 activates ERK1/2 but not Ca^2+^ release^4,12^. Intriguingly, synthetic peptides, such as SLIGKV-NH_2_, SLIGRL-NH_2_, and 2-furoyl-LIGRL-NH_2_, mimic the TL sequence, binding to PAR2 and activating the receptor independently of protease cleavage^4,13^. The ability of various cleavage sites to elicit distinct downstream signaling pathways enables the design of biased peptide ligands that selectively activate one pathway over another, thereby facilitating more precise medical treatments with fewer unwanted side effects. Indeed, a subset of activating peptides has been found to induce specific PAR2-mediated signaling responses. For example, DF253 (2f-LAAAAI-NH_2_) has been demonstrated to trigger PAR2-mediated Ca^2+^ release without inducing ERK1/2 phosphorylation, whereas AY254 (Isox-Cha-Chg-Chg-A-R-NH_2_) acts as an ERK-biased agonist^12^.

The crystal structures of antagonist vorapaxar-bound PAR1^14^ and AZ8838-bound PAR2^15^ reveal the architecture of PARs and offer structural insights into PAR antagonist binding and recognition. However, many key questions regarding PAR signaling remain unanswered, including the active PAR conformations, the mechanism by which the peptide ligand TL binds to and activates PARs, and how different protease-generated TL variants induce distinct downstream signaling pathways. In this study, we employed PAR2 as a model system to elucidate the mechanisms underlying TL binding, PAR activation, and G protein coupling selectivity. We present single-particle cryo-electron microscopy (cryo-EM) structures revealing the active conformation of PAR2 in complex with miniG_s/q_ and miniG_13_, revealing the unique binding mode of TL to PAR2. Furthermore, we elucidate the mechanism of receptor activation and identify key determinants of G protein coupling selectivity, enabling us to design a biased peptide ligand that selectively activates G_q_ signaling. The structural insights provided by our investigation lay the groundwork for understanding PAR2 signaling and offer a rational basis for the development of biased ligands targeting PAR2.

## Results

### R36↓S37 cleavage of PAR2 activates G_q_, G_13_, and G_s_ signaling

To mimic trypsin cleavage of PAR2 between R36 and S37, we removed residues 2–36 of PAR2 and allowed native methionine aminopeptidase (MAP) to remove the N-terminal methionine residue via a process termed N-terminal methionine processing that occurs naturally across prokaryotes and eukaryotes^16^. We employed a bioluminescence resonance energy transfer (BRET) assay consisting of G protein-based tricistronic activity sensors (BRET0)^17^ to quantitatively assess the constitutive activity of GPCR. This approach allowed us to measure the intrinsic activity of the PAR2 constructs. Compared with the pcDNA3 vehicle control, the N-terminally truncated PAR2 (residues 37–397) exhibited strong intrinsic activity in stimulating G_q_, G_13_, and G_s_ signaling but minor self-activity in activating G_i_ signaling (Supplementary Fig. S1a), which is consistent with previous findings^4,6^. On the other hand, the SLIGKV TL has been reported to be incapable of inducing G_s_ signaling by full-length (FL) PAR2^6^. We then examined the effect of TL addition on FL PAR2 signaling in our BRET system. Stimulation with SLIGKV (TL) activated G_q_, G_13_, and G_i_ signaling but had no effect on G_s_ signaling (Supplementary Fig. S1b), which is consistent with previous reports. The observed difference between TL stimulation of FL PAR2 and self-activated truncated PAR2 may be due to the higher coupling efficiency of the endogenous TL peptide that is covalently linked to the N-terminus of the truncated receptor. Notably, FL PAR2 also displays considerable intrinsic G_q_ and G_13_ signaling activity, albeit slightly lower than that of 37–397 PAR2, suggesting the potential proteolytic cleavage of FL PAR2 within the cellular milieu. Both surface expression assays and confocal imaging revealed similar surface expression levels of the Δ(1–36) and FL receptors (Supplementary Figs. S3i, S6a). We also observed the intracellular localization of PAR2 in the confocal images, which may have resulted from transient receptor expression. Nevertheless, the plasma membrane expression of FL PAR2 is comparable to that of the PAR2 Δ(1–36) truncation construct. Given the heightened intrinsic activation of the G_q_ and G_13_ signaling pathways, we decided to obtain the structures of PAR2 in complex with G_q_ and G_13_.

### Overall structures of the PAR2/G_q_ and PAR2/G_13_ complexes

To increase the stability of the complexes, we employed a NanoBiT tethering approach^18^ for their assembly. Specifically, the C-terminus of PAR2 (residues 37–377) was fused with the larger component of NanoBiT (LgBiT) (Supplementary Fig. S1c), while the C-terminus of G protein β subunit 1 (Gβ_1_) was fused with the newly developed high-affinity smaller component of NanoBiT (HiBiT). Coexpression of LgBiT-fused PAR2, Gα, Gγ_2_, and HiBiT-fused Gβ_1_ was achieved in Sf9 cells as described in the Materials and Methods section. For the PAR2/G_q_ complex, a miniGα_s/q_^19^ construct, which contains the crucial G_q_ sequence for receptor engagement, was utilized, whereas for the PAR2/G_13_ complex, a miniGα_i/13_^20^ construct containing the N-terminal region of Gα_i2_ (residues 1–15) was employed. Additionally, a single-chain variable fragment (scFv16)^21^ targeting the N-terminus of Gα_i_ was incorporated into the assembly of the PAR2/miniGα_i/13_ complex, and Nb35 was used in the PAR2/miniG_s/q_ complex to introduce additional features for cryo-EM determination and enhance stability, as outlined in the Materials and Methods section. The use of mini-G proteins facilitates the structural determination of GPCR/G protein complexes, and numerous studies have demonstrated that the engagement conformations of mini-G proteins are in agreement with those of native G proteins^22,23^. Therefore, for convenience in comparison, we designated the PAR2/miniG_s/q_ and PAR2/miniG_i/13_ complexes as PAR2/G_q_ and PAR2/G_13_, respectively. The size exclusion column chromatography profiles of the purified complexes exhibited well-separated peaks (Supplementary Fig. S1d).

**Fig. 1:**
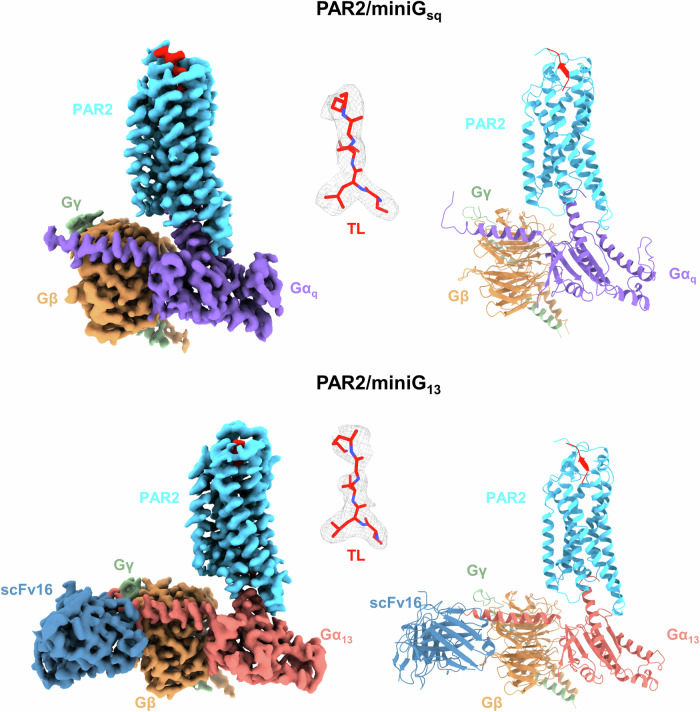
Overall structures of the PAR2/G protein complexes. Left panel, orthogonal views of the cryo-EM density maps; right panel, models of the complexes with the same view and color scheme as shown in the left panel.

The structures of the PAR2/G_q_ and PAR2/G_13_ complexes were elucidated through single-particle analysis by cryo-EM, and resolutions of 3.25 Å and 3.2 Å, respectively, were achieved by the gold-standard criterion of Fourier shell correlation (FSC) = 0.143 (Table 1; Supplementary Fig. S2a, b). Overall, the structures of the PAR2/G_q_ and PAR2/G_13_ complexes closely resemble canonical GPCR/G protein complex structures, wherein the G protein primarily utilizes its α subunit to interact with the receptor (Fig. 1). The receptor sides of the complexes exhibit well-defined densities, which allowed us to model residues 57–359 on the map. Notably, the intracellular loop 2 (ICL2, 182–185) of PAR2 in the G_q_ complex is intact, whereas it is missing in the G_13_ complex because of its flexibility. The TL (residues 37–42) within the ligand-binding pocket is clearly discernible. In the PAR2/G_q_ complex, the density of the Nb35 region is notably lower than that in other parts of the complex (Supplementary Fig. S3a). Consequently, we opted not to include Nb35 in the complex model.

**Table 1:** Cryo-EM data collection and refinement statistics.

	PAR2/miniG_s/q_	PAR2/miniG_13_
	EMD-60243	EMD-60244
	8ZMD	8ZME
**Data collection and processing**		
Magnification	130,000	130,000
Voltage (kV)	300	300
Electron exposure (e^−^/Å^2^)	60	60
Defocus range (µm)	1.2–2.2	1.2–2.2
Pixel size (Å)	1.1	1.1
Symmetry imposed	C1	C1
Initial particle image (no.)	1,569,710	1,354,297
Final particle image (no.)	280,638	176,492
Map resolution (Å)	3.25	3.2
FSC threshold	0.143	0.143
Refinement		
Initial model used (PDB code)	AF-P55085-F1-model_v2	AF-P55085-F1-model_v2
Model resolution (Å)	NA	NA
Map sharpening *B* factor (Å^2^)	−154.7	−151.1
Model composition		
Non-hydrogen atoms	7012	8691
Protein residues	927	1143
Ligands	0	0
*B* factor (Å^2^)		
Protein	89.76	87.59
Ligand	0	0
RMSD		
Bond length (Å)	0.007	0.008
Bond angles (°)	0.804	1.151
Validation		
MolProbity score	1.61	1.36
Clashscore	5.6	2.46
Poor rotamers (%)	0	0
Ramachandran plot		
Favored (%)	95.63	95.29
Allowed (%)	4.37	4.71
Disallowed	0	0

### TL–receptor interactions

**Fig. 2:**
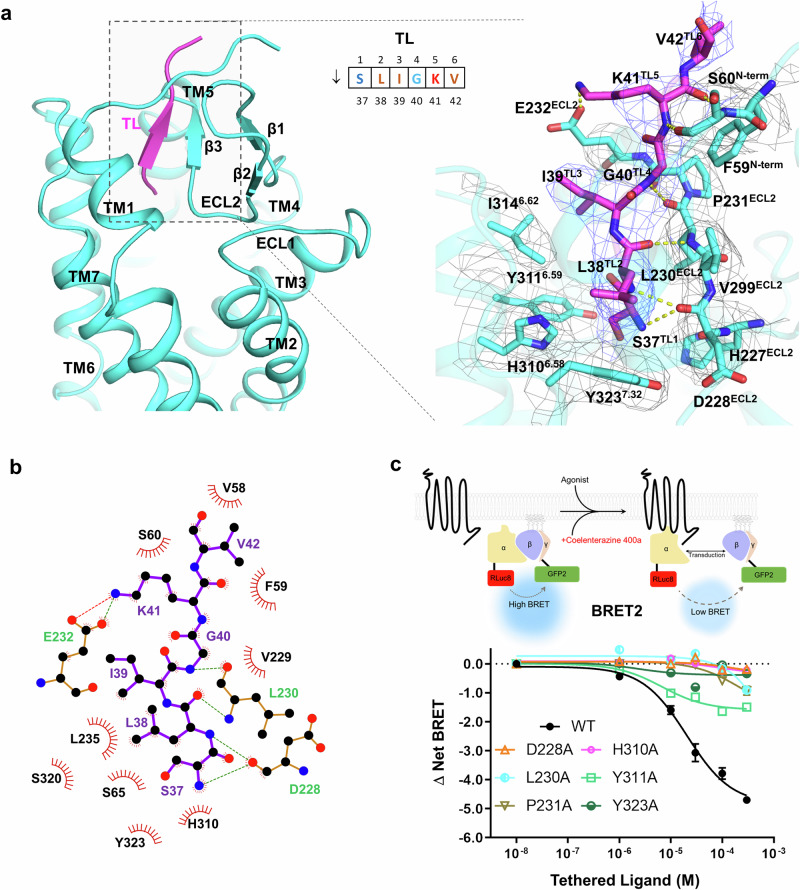
PAR recognition of TL and TL binding. **a** Overall position of TL and the details of TL binding. **b** Schematic map of the TL/receptor interaction. The green dashed line indicates a hydrogen-bonding interaction, and the red dashed line indicates a salt bridge interaction. **c** BRET2 G_13_ dissociation assay of PAR2 mutants. Upper panel, schematic diagram of the BRET2 assay; lower panel, analysis of PAR2 via the BRET2 G_13_ dissociation assay. The data are presented as the mean values ± SD; *n* = 3 independent experimental replicates. The results of the statistical evaluation are listed in Supplementary Table S1.

The receptor sides of the PAR2/G_q_ and PAR2/G_13_ complexes are very similar, with a root mean square deviation (RMSD) of 0.47 Å over 294 Cα pairs (Supplementary Fig. S3b). Because the overall density distribution of the receptor is slightly better in the PAR2/G_13_ complex than in the PAR2/G_q_ complex, particularly in the extracellular region, we used the receptor of the PAR2/G_13_ complex as a model to study the TL/receptor interaction. To provide better insight into how the TL interacts with the receptor, we renumbered the residue (S37) immediately following trypsin cleavage as residue 1 of the TL, the second residue after the cleavage site as residue 2, and so forth (Fig. 2a, upper middle panel). TL occupies the orthosteric pocket of PAR2 and adopts a β-strand conformation to interact with residues 227–231 of extracellular loop 2 (ECL2) (Fig. 2a; Supplementary Fig. S3c). Specifically, the backbone amide groups of S37^TL1^ and L38^TL2^ form hydrogen-bonding interactions with the backbone carbonyl groups of D228^ECL2^ and L230^ECL2^, respectively. At the tail of the TL, the backbone of K41^TL5^ forms hydrogen bonds with the backbone of S60^N-term^, whereas the side chain of K41^TL5^ forms a salt bridge with E232^ECL2^ (Fig. 2a, b). In addition to these direct hydrogen-bonding interactions, S37^TL1^ is surrounded by the polar residues H227^ECL2^, Y323^7.32^, H310^6.58^, and Y311^6.59^. I39^TL3^ forms hydrophobic interactions with I314^6.62^ (Fig. 2a, right panel). We also observed that residues of P231^ECL2^ and V229^ECL2^ play pivotal roles in maintaining the conformation of the β-strand for TL interaction (Fig. 2a, b). An antiparallel β-sheet formed by β1 and β2 may also contribute to the proper folding of the entire ECL2 to engage with the TL (Fig. 2a, left panel). Surface electrostatic analysis of PAR2 indicated that the TL is positively charged, whereas the surrounding surface of the upper pocket is negatively charged (Supplementary Fig. S3d).

We utilized synthetic peptides to mimic the trypsin-revealed TL, enabling functional analysis of PAR2 in a dose-dependent manner (see details in the Materials and Methods section). We used the N-terminal deletion construct of PAR2 (Δ1–48) as the backbone of the receptor to support downstream signaling, as it has lower intrinsic activity than the FL receptor does (Supplementary Fig. S1a). Surface expression assays revealed that the surface expression level of PAR2 (Δ1–48) was similar to that of the wild type (WT) (Supplementary Fig. S3i). On the peptide side, we chose the SLIGRL-NH_2_ peptide (mouse), which differs from SLIGKV-NH_2_ (human) by only the last two residues, as previous studies have shown that the mouse ligand has much higher inducing activity than the human ligand does^13,24^. In the BRET2 assay of the G_13_ sensor, Rluc8 and GFP2 were used as biosensors to measure the separation of Gα from Gβγ^25^ (Fig. 2c, upper panel), and the D228^ECL2^A, L230^ECL2^A, P231^ECL2^A, Y323^7.32^A, H310^6.58^A, and Y311^6.59^A mutants exhibited strongly decreased receptor activity (Fig. 2c, lower panel; Supplementary Table S1), which is consistent with the structural observations of the TL/receptor interaction. Although D228^ECL2^ and L230^ECL2^ interact with TL through their main chains, the carboxyl group of D228^ECL2^ forms a polar interaction with Y323^7.32^, and L230^ECL2^ engages in a hydrophobic interaction with I216^ECL2^. Mutation of these residues to alanine would disrupt both the polar and hydrophobic interactions mentioned above, likely altering the positions of their main chains and consequently impairing TL binding (Supplementary Fig. S3g). We further performed molecular dynamics (MD) simulations to examine the interactions between D228 and Y323, as well as between L230 and I216. The simulation results revealed a stable hydrogen-bonding interaction between D228 and Y323 and a persistent hydrophobic interaction between L230 and I216 (Supplementary Fig. S8), whereas these interactions were lost with the D228A and L230A mutants. Additionally, we observed that residues H227^ECL2^A and V229^ECL2^A of β3, which may not directly participate in the interaction with TL, also decreased receptor activity (Supplementary Fig. S3h and Table S1). Surface expression assays revealed that these mutants were expressed at similar levels compared to the WT receptor (Supplementary Fig. S3i). The endogenous expression of PAR2 in HEK293 cells has been reported^26^; to rule out the possible interference of endogenous PAR2 in AD293 cells in the BRET assay, we examined the response to an empty vector (pcDNA3). The data revealed that there was no TL-mediated induction of G_q_ or G_13_ signaling in the BRET assays (Supplementary Fig. S6b). Furthermore, we fused LgBiT to the C-terminus of PAR2 (tag to receptor) and SmBiT to Gβ and examined how key mutations affect TL binding via a NanoBiT assay in AD293 cells. All the TL interface mutants failed to induce receptor activation (Supplementary Fig. S6c), which is consistent with our previous BRET2 assay results (Fig. 2c). To further exclude potential interference from endogenous Gα proteins in the BRET assay, we developed a modified BRET assay in which the C-terminus of PAR2 was fused to cpVenus (Supplementary Fig. S9a), while Nluc was inserted into the flexible region between αB and αC of Gα, as described in the study by Schihada^17^. Upon ligand addition, PAR2-cpVenus and Gα-Nluc are brought into close proximity, generating a fluorescence signal and thereby avoiding interference from endogenous receptors or G proteins. Consistent with this design, TL stimulation activated PAR2-cpVenus, promoting the recruitment of Gα_q_-Nluc or Gα_13_-Nluc (Supplementary Fig. S9b). No activation was detected in the pcDNA control, confirming that the observed signal originated from exogenous PAR2 rather than endogenous receptors.

The insertion of TL into PAR2 is nearly vertical. Upon examination of available peptide receptor structures, we found that the peptide ligands of AT1R^27^, CCKAR^28^, FPR2^29^, and NTS1R^30^ interact with their receptors in a similar manner; however, these peptides are inserted into receptors to a greater depth than the TL of PAR2 (Supplementary Fig. S3e). Interestingly, the peptide ligands of NPY1R^31^, μOR^32^, ETBR^33^, and APJR^34^ also bind to their receptors perpendicularly; however, they insert in the form of a helix (Supplementary Fig. S3f).

### Receptor activation

**Fig. 3:**
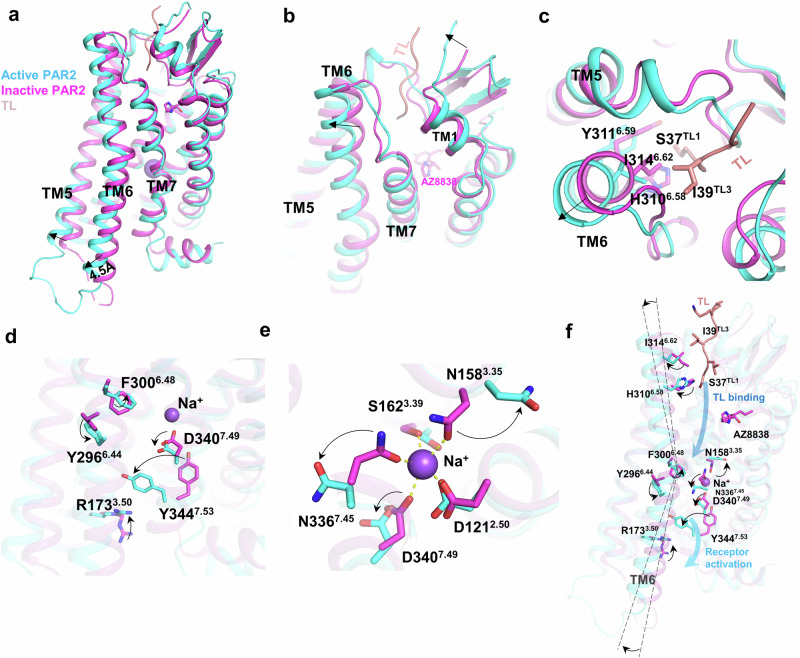
PAR2 activation. **a** Comparison of the overall structure of active PAR2 with that of inactive PAR2 (AZ8838-bound, PDB: 5NDD). **b**, **c** Overall (**b**) and detailed (**c**) conformational changes that occur at the top of the receptor (extracellular side). **d** Conformational changes in the key residues of conserved motifs. **e** Conformational changes in the ion lock upon receptor activation. **f** Representative changes in the complete activation procedure from the top to the bottom of PAR2.

Very interestingly, while TL only occupied the upper portion of the orthosteric pocket of PAR2, the antagonist AZ8838 occupied the bottom left of the pocket (Supplementary Fig. S4a). Compared with the antagonist AZ8838-bound PAR2^15^ structure, a notable conformational change was observed with the TL-bound receptor wherein TM6 on the intracellular side moved outward (Fig. 3a). Although the outward movement (4.5 Å, measured by the Cα of K283^6.31^) is not as drastic as that of other class A GPCRs, it facilitates the opening of the intracellular cavity of PAR2 to allow Gα_q_ or Gα_13_ to engage with the receptor. On the extracellular side, we also observed a small outward movement of TM6 and an inward movement of the N-terminal loop upon TL binding (Fig. 3b). Notably, H310^6.58^ and I314^6.62^ are pushed away by S37^TL1^ and I39^TL3^, respectively, upon TL binding, which forces the outward movement of TM6 on the extracellular side (Fig. 3c).

The bending of TM6 is a characteristic of GPCR activation, which is supported by the rearrangement of the extracellular side of TM6, specifically a twist of F300^6.48^ on the hinge, which coordinates with Y296^6.44^ to stabilize this bent conformation (Fig. 3d). In class A GPCRs, the ion lock effect, which is generally mediated by a sodium ion, locks the center core of the receptor into a tight and compact conformation and is a key mechanism for receptor inactivation^15,35–37^. The bending of TM6 caused a shift of the carboxyl group of D340^7.49^ toward TM6 (Fig. 3d). Concurrently, the carbonyl groups of N336^7.45^ and N158^3.35^ sway toward TM6 and TM2, respectively, causing the collapse of the ion lock (Fig. 3e). Consequently, the collapse of the ion lock allows Y344^7.53^ of the NPxxY motif to shift significantly toward the center of the intracellular cavity. This is accompanied by the collective movement of R173^3.50^ of the DRY motif (Fig. 3d), facilitating full receptor activation. The entire activation process of PAR2 is summarized in Fig. 3f. The binding of TL initiates the outward movement of TM6 on the extracellular side to stabilize its bent conformation, followed by the collapse of the ion lock, ultimately leading to the complete opening of the intracellular cavity for G protein engagement.

### G_q_ and G_13_ engagement and selectivity

**Fig. 4:**
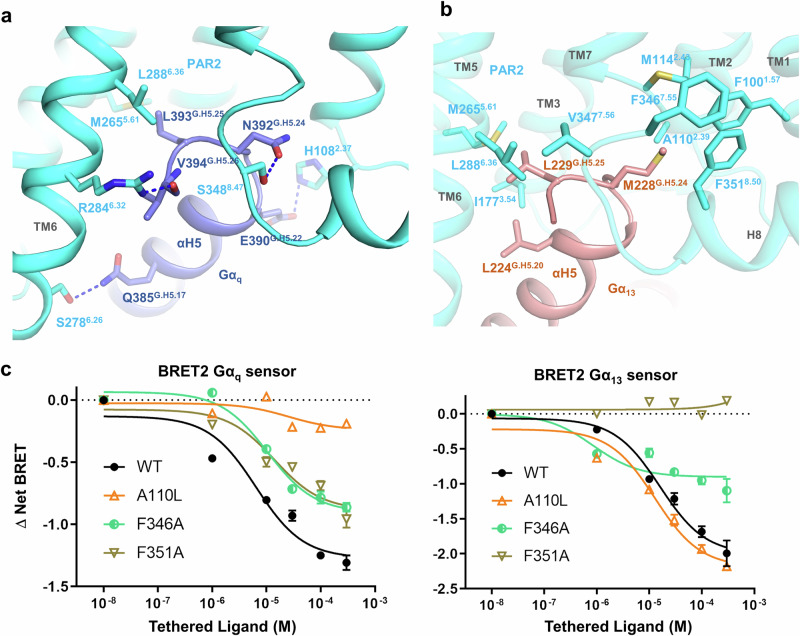
Engagement of G_q_ and G_13_ with PAR2. **a** G_q_ engagement with PAR2. **b** G_13_ engagement with PAR2. **c** BRET2 G_q_ and G_13_ dissociation assays with PAR2 mutants. The data are presented as the mean values ± SD; *n* = 3 independent experimental replicates. The results of the statistical evaluation are listed in Supplementary Table S2.

The overall architectures of the PAR2/G_q_ and PAR2/G_13_ complexes are highly similar, with slight variations observed in the orientations of αN (Supplementary Fig. S4c). Our previous studies on receptors capable of coupling to multiple G proteins suggest that polar interactions play a crucial role in G_q_ and G_s_ engagement, whereas hydrophobic interactions are the main driving force for G_i_ and G_13_ engagement because of the distribution of hydrophobic and hydrophilic residues at the far end of αH5^38,39^. Consistent with this notion, we observed that polar interactions play crucial roles in the PAR2/G_q_ interaction; specifically, the backbone carbonyl group of V394^G.H5.26^ forms a hydrogen bond with R284^6.32^, and N392^G.H5.24^, E390^G.H5.22^ and Q385^G.H5.17^ form hydrogen-bonding interactions with S348^8.47^, H108^2.37^ and S278^6.26^, respectively (Fig. 4a). We also observed hydrophobic interactions between αH5 (L393^G.H5.25^) and the TM3-TM5-TM6 region (M265^5.61^ and L288^6.36^).

Conversely, the engagement of PAR2/G_13_ is predominantly driven by hydrophobic interactions in two patches. The first patch is on the TM6, TM5 and TM3 side; specifically, L224^G.H5.20^ and L229^G.H5.25^ form hydrophobic interactions with L288^6.36^, M265^5.61^, I177^3.54^ and V347^7.56^ (Fig. 4b). The second patch is on the TM7, H8, TM1 and TM2 side, where M228^G.H5.24^ notably inserts into a hydrophobic pocket formed by residues F346^7.55^, F351^8.50^, F100^1.57^, A110^2.39^ and M114^2.43^ (Fig. 4b; Supplementary Fig. S4d). We are particularly interested in the second patch of hydrophobic interactions, as it is missing in the G_q_ complex, where the hydrophobic residue M228^G.H5.24^ is replaced by a polar residue N392^G.H5.24^ (Fig. 4a). We speculated that the hydrophobic interaction between M228^G.H5.24^ and the hydrophobic pocket, termed the “methionine pocket”^40^, is the key determinant of the G_13_ selectivity of PAR2. To test this hypothesis, we mutated phenylalanine residues F346^7.55^ and F351^8.50^ to the small residue alanine (F346^7.55^A and F351^8.50^A) to decrease the hydrophobicity of the pocket. Interestingly, in the BRET assay, both F346^7.55^A and F351^8.50^A exhibited strongly decreased G_13_ activity but had minimal effects on G_q_ activity (Fig. 4c; Supplementary Table S2). In contrast, mutation of the “methionine pocket” border residue A110^2.39^ to leucine (A110^2.39^L) had no effect on G_13_ activity but almost completely abolished G_q_ activity, likely due to the increased size of the hydrophobic side chain, which caused clashes with N392^G.H5.24^ of Gα_q_ (Fig. 4a, c). Notably, all the mutants exhibited similar surface expression levels (Supplementary Fig. S3i).

### Biased agonism and the design of a G_q_-biased peptide ligand

**Fig. 5:**
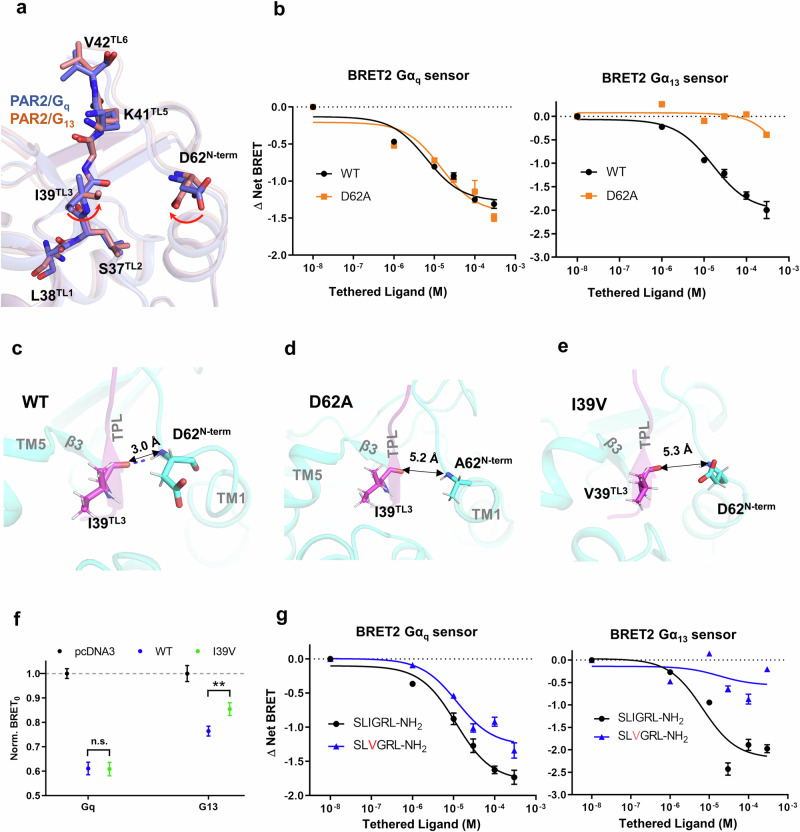
Biased agonism of PAR2. **a** Comparison of the conformations of the TL/receptor between the PAR2/G_q_ and PAR/G_13_ complexes. **b** BRET2 G_q_ and G_13_ dissociation assays with the D62A mutant and synthetic WT peptide. The data are presented as the mean values ± SD; *n* = 3 independent experimental replicates. The results of the statistical evaluation are listed in Supplementary Table S2. **c**–**e** Snapshots of the MD simulation trajectories of WT PAR2 (**c**) and the D62A (**d**) and I39V (**e**) mutants. **f** BRET0 assay to determine the intrinsic activity of the Δ(1–36) truncation mutants of PAR2. The data are presented as the mean values ± SD; *n* = 3 independent experimental replicates. n.s. not significant, **P < 0.01, two-sided *t-*test. **g** BRET2 G_q_ and G_13_ dissociation assays with synthetic peptide ligands. The data are presented as the mean values ± SD; *n* = 3 independent experimental replicates. The results of the statistical evaluation are listed in Supplementary Table S3.

We next asked whether a small change in the ligand-binding pocket of PAR2 may alter the G protein selectivity of this receptor, as this could provide useful information for designing biased ligands aimed at reducing undesirable side effects. We first compared TL binding in the PAR2/G_q_ complex and the PAR2/G_13_ complex. The binding poses of TL in the G_q_ and G_13_ complexes are very similar, with minor differences noted: (1) the carboxyl group of D62^N-term^ faces TL in the G_13_ complex but turns away in the G_q_ complex; and (2) the side chain of I39^TL3^ faces D62^N-term^ in the G_13_ complex but turns away in the G_q_ complex (Fig. 5a). On the basis of the above observations, we speculated that D62^N-term^ may play a role in the G protein selectivity of PAR2. Interestingly, the D62^N-term^ A mutation almost completely abolished G_13_ activity but only had a minor effect on G_q_ activity in the BRET2 assay, which aimed to measure the dose response with the synthetic SLIGRL-NH_2_ peptide (Fig. 5b; Supplementary Table S2). Notably, the surface expression level of the D62^N-term^A mutant was similar to that of the WT receptor (Supplementary Fig. S3i). In addition to the BRET2 assay, wherein Rluc8 and GFP2 were used as the biosensor system^25^, we also used the BRET0 assay, wherein the biosensor system was constructed by Nluc and cpVenus fused at different positions of Gα and Gγ^17^. We further used the NanoBiT assay^11,41^, in which the large part of NanoBiT was fused to Gα and the small part of NanoBiT was fused to Gβ. All these assays revealed that D62^N-term^A is clearly biased toward G_q_ signaling and exhibits minimal G_13_ activity (Supplementary Fig. S5a).

Since cryo-EM structures capture only snapshots of receptor conformation, we used MD simulations to understand the underlying mechanism by which D62^N-term^A selectively blocks G_13_ activity while retaining most G_q_ activity. We speculated that D62^N-term^ may somehow interact with TL to pull it toward TM1. Despite the lack of a direct interaction of I39^TL3^/D62^N-term^ in the cryo-EM structure of PAR2, we observed the constant association between TL and the N-terminal region of TM1 owing to a hydrogen bond between the backbone amide group of D62^N-term^ and the backbone carbonyl group of I39^TL3^ in the MD simulations with WT PAR2 (Fig. 5c; Supplementary Fig. S7a and Video S1). In contrast, in the MD simulations with the D62^N-term^A mutant of PAR2, A62^N-term^ is far from I39^TL3^ (~5.2 Å), and there were no hydrogen-bonding interactions between A62^N-term^ and I39^TL3^ throughout the whole simulation (Fig. 5d; Supplementary Fig. S7b and Video S2). These findings suggest that the closer proximity of TL with the N-terminus of TM1, mediated by I39^TL3^/D62^N-term^, is crucial for G_13_ coupling.

We then investigated whether mutation to I39^TL3^ could separate G_13_ activity from G_q_ activity. Similar to the D62^N-term^A mutant, in the MD simulations, the I39^TL3^V mutant is positioned far from the N-terminus of TM1 and loses its ability to form hydrogen bonds with D62^N-term^ (Fig. 5e; Supplementary Fig. S7c and Video S3). In contrast, most of the hydrogen-bonding interactions between TL and the N-terminus of TM1 were preserved with the I39^TL3^L mutant (Supplementary Fig. S7d and Video S4). Interestingly, in a BRET0 assay designed to measure the intrinsic activity of the self-activated 37–397 construct of PAR2 (mimicking trypsin cleavage of PAR2), we observed clear separation of G_13_ activity from G_q_ activity with the I39^TL3^V mutant (Fig. 5f). Notably, the surface expression level of the I39^TL3^V mutant was similar to that of the WT receptor (Supplementary Fig. S3i). Conversely, the I39^TL3^L mutant retained most of the activity of WT PAR2, resulting in little separation of G_13_ activity from G_q_ activity (Supplementary Fig. S4e). Other I39^TL3^ mutants, such as I39^TL3^D, I39^TL3^A, and I39^TL3^F, did not clearly show biased signaling. Notably, the surface expression levels of all the mutants were similar to those of the WT receptor (Supplementary Fig. S3i). We then asked whether a valine mutation at the third residue of the TL peptide could separate G_13_ activity from G_q_ activity and thus serve as a biased ligand. For this purpose, we synthesized the SLVGRL-NH_2_ peptide, which indeed maintained most of the G_q_ activity of the WT peptide but lost most of the G_13_ activity in the BRET2 assay (Fig. 5g; Supplementary Table S3). To validate that the I39^TL3^V mutant of the synthetic PAR2 TL is a biased agonist, we employed both BRET0 and NanoBiT assays to assess the activity of the synthetic SLVGRL-NH_2_ peptide. Both assays revealed that the I–V mutation of residue 39 of the synthetic PAR2 TL exhibited a strong bias toward G_q_ signaling, with minimal G_13_ activity (Supplementary Fig. S5b).

## Discussion

Despite playing crucial roles in the inflammatory response and pain sensation, structural knowledge of PARs is limited primarily to the inactive conformations of PAR1 and PAR2. The mechanism by which proteolytic cleavage of the N-terminus activates these receptors remains unclear. In this study, we utilized an N-terminally truncated PAR2 construct and native N-terminal methionine processing to mimic trypsin proteolysis of PAR2, aiming to gain structural insights into TL binding and PAR2 activation. To our surprise, the orthosteric pocket of PAR2 is shallower than that of most class A GPCRs. This differs from aGPCRs, where the stalk peptide inserts deep into the ligand-binding pocket. The activation of PAR2 is initiated by the outward movement of TM6 on the extracellular side, which is propelled by the binding of TL to the β3-sheet structure of ECL2. This is followed by the rearrangement of the receptor core to disrupt the ion lock, thereby releasing TM6 to facilitate G protein binding. Unexpectedly, compared with that of other receptors, the outward movement of TM6 upon activation is minor, and we wondered whether this is a feature unique to PAR2 or a general characteristic of closely related receptors. PAR1 shares the highest degree of homology with PAR2. While our manuscript of PAR2 was under review, the active conformation of PAR1 in complex with G_q_ was reported in another study^42^. Additionally, FFAR2, FFAR3, and FFAR1 are phylogenetically similar to PAR2. A comparison of the outward movement of TM6 between PAR2, PAR1, FFAR2, FFAR3, and FFAR4 revealed that these receptors exhibit similar TM6 outward movements (Supplementary Fig. S4b). In contrast, β_2_AR, which was used as a control, displayed much greater outward movement of TM6 upon activation. These findings suggest that the small outward movement of TM6 might be an intrinsic property of PAR2 and these closely related receptors.

In our study, we observed that the TL engages extensively with ECL2, particularly the β3 strand (residues 227–231), to activate the receptor. Mutation of key residues in ECL2 significantly reduces receptor activity, which is consistent with previous findings that ECL2 plays a critical role in mediating TL binding^43,44^. This TL–receptor interaction pattern appears to be conserved across PAR family members, as mutational studies of PAR1, PAR2, and PAR4 have similarly implicated ECL2 in ligand engagement^45–48^. Our structural study also aligns well with previous structure‒activity relationship studies of PAR2^12,13,49–51^. A previous study revealed that the SLAGRL-NH_2_ peptide has significantly lower efficacy^13^, but we demonstrated that the G_q_ signaling activity of SLVGRL-NH_2_ is similar to that of SLIGRL-NH_2_ in BRET0, BRET2, and NanoBiT assays (Fig. 5g; Supplementary Fig. S5b). We further used an NFAT-RE_luciferase assay to show that the activity of the SLVGRL-NH_2_ peptide is similar to that of SLIGRL-NH_2_ in inducing G_q_ signaling (Supplementary Fig. S6d). More recently, a study reported the structure of PAR2 bound to both TL and the selective small-molecule agonist GB88 in complex with G_q_^52^. While both their TL-bound PAR2/G_q_ complex and our complex show a similar receptor architecture (Supplementary Fig. S9c), a closer comparison reveals key differences. Their structure resolves only the first three residues of the TL and lacks structural information on the N-terminal loop preceding TM1. In contrast, our structure captures the FL of the TL and its interaction with the N-terminal loop (Supplementary Fig. S9d), providing deeper insight into the mechanism of TL–receptor engagement.

Interestingly, protease cleavage at different sites of the N-terminus of PAR2 results in distinct signaling activities. We attempted to obtain structures of the Δ(1–56) and Δ(1–67) PAR2 constructs complexed with G proteins to understand the activation of PAR2 by CS (E56↓T57 cleavage) and NE (S67↓V68 cleavage). However, these complexes were not stable enough to allow for further structural determination. On the basis of our structural observation of trypsin activation of PAR2 (37–377), the N-terminal region of PAR2 (57–70) is important for TL binding and receptor activation. Disruption of this region causes a significant rearrangement of the receptor conformation and thereby destabilizes the receptor. Therefore, the structural basis of the activation of PAR2 by CS and NE remains unclear and requires further exploration.

A direct comparison of G_q_ coupling to PAR2 with G_13_ coupling suggested that a unique hydrophobic interaction between the last methionine residue of Gα_13_ αH5 (M228^G.H5.24^) and the hydrophobic pocket composed of TM7-H8-TM1-TM2 is the key determinant of G_13_ selectivity, and this observation is supported by a mutagenesis study in which a decrease in the hydrophobicity of the pocket reduced G_13_ activity but had no effect on G_q_ activity. Thus, does the upper part of the receptor, which hosts ligand binding, also have the ability to separate G_13_ signaling from G_q_ coupling?

**Fig. 6:**
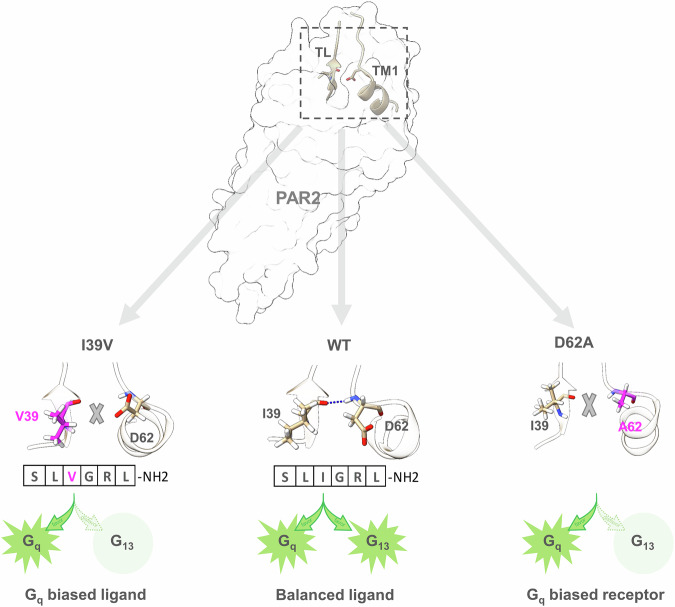
Schematic diagram of the design of the biased PAR2 ligand. The diagram shows that disrupting the hydrogen bond between I39 and D62 — either through mutation of the TL peptide (I39V, left panel) or the TM1 residue (D62A, right panel) — results in G_q_-biased signaling.

Our structural analysis revealed subtle differences in the N-terminus of TM1 (D62^N-term^) and the third residue of TL (I39^TL3^) between the G_q_ complex and the G_13_ complex. Aided by MD simulations, which provide a dynamic interaction landscape that is sometimes compromised by the snapshots of structural methods (e.g., crystallization and cryo-EM), we determined that the hydrogen-bonding interaction between I39^TL3^ and D62^N-term^ is crucial for G_13_ activity but not essential for G_q_ signaling. Disrupting this hydrogen bond, either by replacing D62^N-term^ with alanine (D62^N-term^A) or by substituting I39^TL3^ with valine (I39^TL3^V), separates G_13_ activity from G_q_ activity, thereby offering an opportunity to design a biased ligand that selectively activates G_q_ signaling (Fig. 6). To understand how these small changes in I39^TL3^ and D62^N-term^ affect PAR2 signaling, we examined the specific interactions of these residues. With respect to D62^N-term^, in the PAR2/G_q_ complex, the carboxyl group forms a hydrogen bond with the backbone amide group of E63^N-term^ (Supplementary Fig. S9e), whereas in the PAR2/G_13_ complex, it forms a hydrogen bond with S65^N-term^ (Supplementary Fig. S9f). Mutation of D62^N-term^ to alanine (D62^N-term^A) disrupts its association with the N-terminus of TM1 in both the G_q_ and G_13_ complex, destabilizing the conformation of the N-terminus of TM1. With respect to I39^TL3^, the longer methyl group of isoleucine allows for hydrophobic interactions with L235^5.31^ and I314^6.62^ (Supplementary Fig. S9g), stabilizing peptide conformation. In contrast, the I39^TL3^V mutant has a shorter side chain, preventing it from forming these hydrophobic interactions. Such subtle changes at this position can decrease the stability of the TL peptide, impacting its ability to form hydrogen bonds with D62^N-term^, as seen in the MD simulations. Compared with the PAR2/G_q_ complex, the PAR2/G_13_ complex appears to be more sensitive to these changes.

Indeed, the synthetic peptide SLVGRL-NH_2_ predominantly activates G_q_ signaling and has a minimal effect on G_13_ signaling. We speculate that the I39^TL3^/D62^N-term^ interaction causes TL to move toward the TM1 side, creating a perturbation on TM1 from the top of the receptor (extracellular side) to the bottom of the receptor (intracellular side). This perturbation could position the bottom side of TM1 (e.g., F100^1.57^), as well as nearby residue F346^7.55^, in the correct position to form the hydrophobic pocket for M228^G.H5.24^ for interaction with αH5 (Fig. 4b), which is the key determinant of G_13_ selectivity, as mentioned earlier. Notably, NE cleavage selectively activates ERK1/2 phosphorylation without inducing Ca^2+^ release, whereas CS cleavage triggers cAMP accumulation only^4,12^. Both of the proteases cleave the receptor near the N-terminal region of TM1, highlighting the crucial role this region plays in G protein selection. While the importance of PAR2-biased signaling has not been thoroughly investigated, and direct connections between ERK signaling and G_13_ or between Ca²⁺ signaling and G_q_ have not been clearly established, our development of a G_q_-biased peptide may not have an immediate application but may simply serve as a proof of concept for designing biased agonists and may hold potential value as research progresses. While our study revealed the activation of PAR via TL, it is interesting to explore how small molecules activate PAR2. For this purpose, we docked AC264613, a potent PAR2-selective agonist, into our model of PAR2. Surprisingly, the top three docking poses show that the conformation of AC264613 mimics that of the N-terminus of TL, particularly the first two residues of TL, to activate the receptor (Supplementary Fig. S10a). Interestingly, these results align well with the binding mode of GB88 (Supplementary Fig. S10b), a partial PAR2 agonist, which is reported in a recent study^52^.

In summary, we solved the cryo-EM structures of PAR2 in complex with G_q_ and G_13_, revealing mechanisms of PAR2 activation and biased agonism. We further designed a G_q_-biased peptide ligand on the basis of our structural discovery. The structural insights from our study lay the groundwork for understanding PAR signaling and serve as a blueprint for designing biased ligands targeting PAR2.

## Materials and methods

### Constructs

The codon-optimized human *PAR2* gene (residues 37–377) was included LgBiT fused to the C-terminus of its encoded protein, after which a tobacco etch virus (TEV) cutting site and 2× maltose-binding protein (MBP) were cloned and inserted into the pFastBac1 baculovirus expression vector. The human Gβ_1_ with a C-terminal HiBiT fusion was cloned and inserted into the pFastBac1 plasmid as described for the VIP1R paper^18^. The mini-Gα_q_ and mini-Gα_13_ constructs were adopted from previous studies on the ghrelin receptor/G_q_ complex^19^ and latrophilin 3/G_13_ complex^20^, respectively. The sequences were codon optimized and synthesized by Langjing Biotech (Shanghai) and inserted into the pFastBac1 vector. The WT human Gγ_2_, a scFv16 that encodes the single-chain variable fragment of mAb16, was cloned and inserted into the pFastBac1 plasmid.

### Protein expression and purification

Recombinant baculoviruses encoding the PAR2-LgBiT-TEV-2MBP, Gα (mini-Gα_s/q_ and mini-Gα_i/13_), Gβ_1_, and Gγ_2_ proteins were coinfected into *Spodoptera frugiperda* (Sf9) cells. For G_13_ complex assembly, scFv16 was coexpressed to stabilize the complex. Cells were cultured at 27 °C and 110 rpm for 48 h at a density of 2 × 10^6^ cells/mL before infection at a ratio of 1:100 (virus volume to cell volume). The cell pellets were resuspended in 20 mM HEPES (pH 7.5), 150 mM NaCl, 10 mM MgCl_2_, 20 mM KCl, 5 mM CaCl_2_, 0.5 mU/mL apyrase, and 20 μg/mL Nb35 for assembly of the G_q_ complex and then homogenized by douncing ~30 times. After 1 h of incubation at room temperature, 0.5% (w/v) lauryl maltose neopentylglycol (LMNG, Anatrace) and 0.1% (w/v) cholesteryl hemisuccinate Tris salt (CHS, Anatrace) were added to solubilize the membrane at 4 °C for 2 h. Then, the lysate was ultracentrifuged at 65,000× *g* at 4 °C for 45 min. The supernatant was loaded onto an amylose column and incubated for 2 h, after which the column was washed with buffer (20 mM HEPES (pH 7.5), 150 mM NaCl, and 0.01% LMNG/0.002% CHS), and the protein was eluted with the same buffer supplemented with 10 mM maltose. After concentration and TEV cleavage overnight at 4 °C, the complex was injected onto a Superdex 200 Increase 10/300 GL gel infiltration column (GE Health Science) that had been pre-equilibrated with buffer containing 20 mM HEPES (pH 7.5), 150 mM NaCl, and 0.00075% (w/v) LMNG, 0.00025% (w/v) glycodiosgenin (GDN, Anatrace), and 0.0002% (w/v) CHS. The corresponding fractions containing the PAR2/G protein complex were concentrated to ~10 mg/mL and snap-frozen for subsequent cryo-EM grid preparation.

### Expression and purification of Nb35

Nanobody-35 (Nb35) bearing a C-terminal His-tag was expressed in the periplasm of *Escherichia coli* BL21 and grown in TB culture medium supplemented with 100 μg/mL ampicillin, 2 mM MgCl_2_, and 0.1% (w/v) glucose at 37 °C and 200 rpm. When the OD600 reached 0.7–0.9, 1 mM IPTG was added to induce protein expression. Induced cultures were grown for 4–6 h at 28 °C. The cells were collected by centrifugation and lysed in a frozen buffer solution (50 mM Tris (pH 8.0), 0.125 mM sucrose, and 0.125 mM EDTA). After lysis, the cell fragments were removed by centrifugation, and Nb35 was purified by nickel affinity chromatography. Purified Nb35 was added to 10% (v/v) glycerol and stored at –80 °C until use.

### Grid preparation and cryo-EM data collection

A protein complex sample at a concentration of ~10 mg/mL was applied onto Cu holey carbon grids (Quantifoil R1.2/1.3) that had been pretreated via glow charging (Quantifoil GmbH). The loaded grids were then rapidly frozen by plunging them into liquid ethane using a Vitrobot Mark IV (Thermo Fisher Scientific). The Vitrobot settings were as follows: a blot force of 10, a blot time of 5 s, a humidity of 100%, and a temperature of 4 °C. After preparation, the grids containing evenly distributed particles embedded in thin ice were inserted into an FEI 300 kV Titan Krios transmission electron microscope equipped with a Gatan Quantum energy filter. Imaging was conducted using a Gatan K2 Summit direct electron detector employing superresolution counting mode, with a pixel size of 0.55 Å at a magnification of 64,000×. The energy filter slit was set to 20 eV. Each image comprised 40 frames, with a total exposure time of 7.3 s and a dose rate of 1.5 e^–^/Å^2^/s (resulting in a total dose of 60 e^–^/Å^2^). The nominal defocus value ranged from –1.2 µm to –2.2 µm.

### Data processing

Initially, the raw movies were downsampled once (to 1.1 Å) and motion-corrected using MotionCor2^53^. Afterward, the contrast transfer function (CTF) parameters were determined using CTFFIND 4.1^54^. Particle selection was conducted using crYOLO^55^, followed by reference-free 2D classification within RELION^56^. The distinct 2D features obtained from this classification were utilized to generate an initial model with the ab initio method of cryoSPARC^57^. This initial model served as a reference for subsequent refinement steps in RELION. 3D classification was then performed, resulting in 3–4 classes. The most suitable class that demonstrated clear secondary structure features was chosen for nonuniform refinement in cryoSPARC. Subsequently, a no-alignment 3D classification was executed in RELION, involving 6–10 classes and imposing a mask on the complex. Bayesian polishing^58^ and additional rounds of nonuniform refinement were conducted to improve the map quality. The resolution of the final map was assessed using the gold-standard FSC criterion at FSC = 0.143. Local resolution assessments were carried out using an integrated program within cryoSPARC.

### Model building

We employed the AlphaFold^59^-predicted structure of human PAR2 (AF-P55085-F1-model_v2) as the initial model to guide the process of model building against the electron microscopy map. This model was docked into the density map using UCSF Chimera^60^. Iterative manual adjustments were carried out in Coot^61^ to refine the models, followed by Rosetta cryo-EM refinement^62^ and Phenix real space refinement^63^ to further improve structural accuracy. To visualize and prepare structural figures, UCSF ChimeraX^64^ and PyMOL (https://pymol.org/2/) were used.

### MD simulations

The cryo-EM structure of TL-bound PAR2 (receptor only) was used as the initial model for the MD simulations. The tethered peptide and the receptor were assigned to different chains. Residues 43–56, which were invisible in the cryo-EM structure, were deleted. The model was prepared and parameterized in CHARMM-GUI^65^. Protonation states of all the titratable residues were assigned at pH 7.0 via Propka^66,67^. Mutagenesis of a single residue was achieved in CHARMM-GUI. The PAR2 model was inserted into a lipid bilayer containing palmitoyl-2-oleoyl-sn-glycero-3-phosphocholine (POPC) and cholesterol at a 4:1 ratio. The membrane had dimensions of 65 Å × 65 Å, with 22.5 Å of water on the top and bottom (resulting in final system dimensions of approximately 65 Å × 65 Å × 120 Å). The ion concentration was set to 0.15 M KCl. The Amber force fields were configured as follows: protein FF19SB, lipid LIPID17, water TIP3P, and ligand GAFF2. Simulations were conducted using the Amber20 package^68^. The system underwent initial energy minimization for the solvent and all the atoms, followed by heating to 300 K over 300 ps and equilibration for 700 ps. Three independent production runs of 200 ns each were subsequently performed with a time step of 2 fs. During the simulations, the particle mesh Ewald algorithm was used to calculate long-range electrostatic interactions, whereas a cutoff of 10 Å was applied for short-range electrostatic and van der Waals interactions. SHAKE algorithm constraints were applied to all the bonds involving hydrogens. The temperature (300 K) and pressure (1 atm) were controlled by the Langevin thermostat and Berendsen barostat, respectively. Each 200 ns simulation was run independently six times. Trajectory analysis and visualization were carried out using VMD, as was video recording^69^.

### BRET2 assays

In accordance with previous methods^25^, AD293 cells were plated in 6-well plates at 700,000–800,000 cells per well. After 1 day of growth at 37 °C with 5% CO_2_, the cells in each well were transfected with 100 ng of pcDNA3-PAR2 (Δ1–48) WT or mutants, 100 ng of Gα-RLuc8, 100 ng of Gβ, and 100 ng of Gγ-green fluorescent protein (Gγ-GFP) plasmids with Lipofectamine 2000 (Thermo Fisher Scientific). After being incubated for 1 day, the cells were collected, pelleted by centrifugation, and suspended in DMEM supplemented with 5% (v/v) FBS. The cell suspensions were dispensed in white 96-well plates at a density of 25,000–50,000 cells per well. One day after being plated, the growth medium was carefully aspirated and replaced immediately with 60 μL of 1× Hank’s balanced salt solution (HBSS) containing 20 mM HEPES (pH 7.4; assay buffer), followed by the addition of 10 μL of freshly prepared 50 μM coelenterazine 400a (Yeasen). After a 5-min equilibration period, the cells were treated with 30 μL of drug for an additional 5 min. The plates were then measured in an EnVision plate reader (PerkinElmer) with 395 nm (RLuc8-coelenterazine 400a) and 510 nm (GFP2) emission filters with integration times of 1 s. The BRET signal was calculated by the ratio of the luminescence intensity at 510 nm (GFP) to that at 395 nm (RLuc), and the Δnet BRET ratio was calculated by subtracting the luminescence intensity of the vehicle control from that of each experimental well (510 nm/395 nm). The data were subsequently plotted as a four-parameter sigmoidal concentration–response curve in GraphPad Prism 8.

### BRET0 assay using tricistronic activity sensors

To measure the self-activation of WT and mutant PAR2 in response to G protein activity, a tricistronic activity sensor assay was performed as previously described^17^. The G protein sensor plasmids were obtained from Addgene (https://addgene.org/Gunnar_Schulte/). To measure constitutive activity, AD293 cells in 6-well plates were cotransfected with 500 ng of WT or mutant PAR2 and 500 ng of the G protein sensor construct (pcDNA-Gα-Nluc/Gβ_3_/cpVenus-Gγ_9_ fusion combination) with Lipofectamine 2000 (Invitrogen). After being incubated for 24 h at 37 °C in a 5% CO_2_ atmosphere, the transfected cells were seeded into white 96-well plates and grown for another 24 h. The cells were subsequently washed with HBSS and incubated with a 1:1000 dilution of furimazine stock solution. After incubation for 3 min at 37 °C, the BRET ratio was measured twice consecutively to assess constitutive receptor activity using an EnVision multimode plate reader (PerkinElmer). Nluc emission intensity was selected using a 450/40-nm monochromator, and cpVenus^173^ emission was selected using a 535/30-nm monochromator with an integration time of 0.3 s in both channels. The ratio of cpVenus/Nluc intensity was calculated for each well and normalized to that of the vehicle control.

For ligand-induced BRET0 measurements, transfected cells were plated in 96-well plates for 24 h, after which the cells were washed with HBSS and incubated with a 1:1000 dilution of furimazine stock solution. After being incubated for 3 min at 37 °C, the cells were treated with the addition of ligand solutions or vehicle control. Plates were then subjected to BRET measurements with an EnVision plate reader (PerkinElmer) at 450/40-nm (Nluc-furimazine) and 535/30-nm to detect ligand-induced changes in the BRET signal. Like in the BRET2 assay, the BRET signal was calculated by the ratio of the intensity at 535 nm (cpVenus) to that at 450 nm (Nluc), and the Δnet BRET ratio was calculated by subtracting the intensity of the vehicle control from that of each experimental well. The data were subsequently plotted as a four-parameter sigmoidal concentration–response curve in GraphPad Prism 8.

### NanoBiT G protein dissociation assay

Ligand-induced G protein activation was measured by a NanoBiT G protein dissociation assay, as described previously^11,41^. Briefly, AD293 cells cultured in 6-well plates were transfected with plasmids encoding a large fragment (LgBiT)-containing Gα_q_ or Gα_13_ subunit (100 ng), a small fragment (SmBiT)-fused Gβ_1_ subunit (500 ng), and the untagged Gγ_2_ subunit with a C68S mutation (500 ng) in combination with the WT PAR2 construct (200 ng). After being incubated for 1 day, the transfected cells were collected, pelleted by centrifugation, and suspended in HBSS containing 0.01% bovine serum albumin (Solarbio) and 5 mM HEPES (pH 7.4; assay buffer). The cell suspension was dispensed in white 96-well plates at a volume of 80 μL per well and mixed with 20 μL of 50 μM coelenterazine native (BIOSTNTH). After incubation for 2 h at RT, the baseline luminescence was measured, after which 20 μL of test compound (6×) was added, and subsequent luminescence measurements were performed to detect ligand-induced changes. Luminescence intensity measured from 3 min to 5 min after compound addition was normalized to the initial intensity, and the net luminescence was calculated by subtracting the luminescence ratio of the vehicle control from the normalized luminescence per well. The G protein dissociation signals were fitted to a four-parameter sigmoidal concentration–response curve (GraphPad Prism 8).

### Measurement of receptor cell surface expression by ELISA

To evaluate the expression levels of WT PAR2 (49–397) and its mutants on the cell surface, AD293 cells were transiently transfected with varying amounts of plasmids encoding target receptors or a vehicle plasmid (pcDNA3) using Lipofectamine 2000 (Invitrogen) in a 6-well plate for 24 h of incubation. Before being seeded into 48-well plates, the plates were coated with a solution of poly-l-lysine at a concentration of 0.02 mg/mL for at least 30 min at 37 °C, and the poly-l-lysine solution was removed by aspiration. Cells were seeded into a 48-well plate at a density of 2 × 10^5^ cells per well and further incubated for 24 h at 37 °C with 5% CO_2_. The cells were then fixed with 4% (w/v) paraformaldehyde for 15 min at room temperature and blocked with DMEM supplemented with 10% (w/v) FBS for another hour. Afterward, the cells were washed and incubated with a mouse monoclonal anti-Flag M2 primary antibody (Sigma‒Aldrich, 1:1,000) at room temperature for 1 h. After the cells were washed, 3,3′,5,5′-tetramethylbenzidine (TMB) solution was added to initiate a color reaction, which was then stopped by adding an equal volume of 3 M H_2_SO_4_. The absorbance at 450 nm was measured using an EnVision multimode plate reader (PerkinElmer).

### Confocal microscopy

PAR2 (FL) and PAR2 (37–397) were fused with GFP, and then 400 ng of PAR2 (FL)-GFP or PAR2 (37–397)-GFP was transfected into AD293 cells with Lipofectamine 2000 at a ratio of 1:2 for ~20 h. Individual confocal images were acquired at identical settings with a 100× oil immersion lens (Zeiss Confocal Microscope LSM880) with Fast Airyscan.

### Docking studies

The docking method performed was similar to that in a previous study^70^. Briefly, the cryo-EM structure of PAR2 (receptor only) was used as the template. The model was prepared and minimized as before. AC264613 was docked in the ligand-binding pocket using the triangle matcher with a London docking score. Refinement was employed on the basis of a rigid receptor and GBVI/WSA docking scoring. The highest scoring poses are shown in Supplementary Fig. S10a.

### Cell-based reporter assay

NFAT-RE reporter assays (Promega) were performed according to the manufacturer’s instructions as described previously^71^. Briefly, AD293 cells were seeded into 24-well plates at a density of 40,000 per well and then transfected with 100 ng of reporter, 10 ng of pcDNA3-PAR2, and 10 ng of phRGtkRenilla plasmids (Promega) with X-tremeGENE HP (Roche) at a 3:1 ratio over DNA after 1 day of growth at 37 °C with 5% CO_2_. Sixteen hours after transfection, the cells were induced with vehicle or 300 μM TL (SLIGRL-NH_2_ or SLVGRL-NH_2_). Six hours after induction, the cells were harvested and lysed by the addition of 1× Passive Lysis Buffer (Promega). The luciferase activity was assessed by the Dual-Glo Luciferase system (Promega). Data were plotted as firefly luciferase activity normalized to Renilla luciferase activity in relative luciferase units (RLUs).

### BRET0 assay for Gα recruitment measurement

To measure the direct interaction between PAR2 and Gα subunits, we developed a new BRET assay. In this assay, the C-terminus of PAR2 was fused with cpVenus, and Nluc was inserted into Gα at the flexible region between αB and αC, as described in a previous study^17^. Briefly, AD293 cells were plated in 6-well plates at 700,000–800,000 cells per well. After 1 day of growth at 37 °C with 5% CO_2_, the cells in each well were transfected with 200 ng of WT PAR2 (Δ1–48) fused with cpVenus to its C-terminus, 200 ng of Gα-Nluc, 500 ng of Gβ, and 500 ng of Gγ plasmids with Lipofectamine 2000 (Thermo Fisher Scientific). After being incubated for 1 day, the cells were collected, pelleted by centrifugation, and suspended in DMEM supplemented with 5% (v/v) FBS. The cell suspension was dispensed in white 96-well plates at a density of 25,000–50,000 cells per well. After being incubated for 24 h at 37 °C in a 5% CO_2_ atmosphere, the transfected cells were seeded into white 96-well assay plates and grown for another 24 h. The cells were subsequently washed with HBSS and incubated with a 1:1000 dilution of furimazine stock solution. After being incubated for 3 min at 37 °C, the cells were treated with the addition of ligand solutions or vehicle control. Plates were then subjected to BRET measurements with an EnVision plate reader (PerkinElmer) at 450/40-nm (Nluc-furimazine) and 535/30-nm to detect ligand-induced changes in the BRET signal. Like in the BRET2 assay, the BRET signal was calculated by the ratio of the intensity at 535 nm (cpVenus) to that at 450 nm (Nluc), and the Δnet BRET ratio was calculated by subtracting the intensity of the vehicle control from that of each experimental well. The data were subsequently plotted as a four-parameter sigmoidal concentration–response curve in GraphPad Prism 8.

## Supplementary information

Supplementary Information

Supplementary Video S1

Supplementary Video S2

Supplementary Video S3

Supplementary Video S4

## Data Availability

All data produced or analyzed in this study are included in the main text or the supplementary figures/tables. The cryo-EM density maps and atomic coordinates have been deposited in the Electron Microscopy Data Bank (EMDB) and Protein Data Bank (PDB) under accession numbers EMD-60243 and 8ZMD for PAR2/G_q_ complex; EMD-60244 and 8ZME for PAR2/G_13_ complex. The MD simulation data were deposited to Zenodo (https://zenodo.org/) under ID: 14242000, 14243031, 14247963, and 14248936 for PAR2 WT, D62A, I39V, and I39L, respectively.
