# Supplementary material for: Structural basis of protease-activated receptor 2 activation and biased agonism
Source: Cell Discov. 2025 Dec 2;11:96. doi: 10.1038/s41421-025-00851-8 (PMC12673148; doi:10.1038/s41421-025-00851-8)
Supplement: Supplementary file 1 — Supplementary Information [file 41421_2025_851_MOESM1_ESM.pdf]

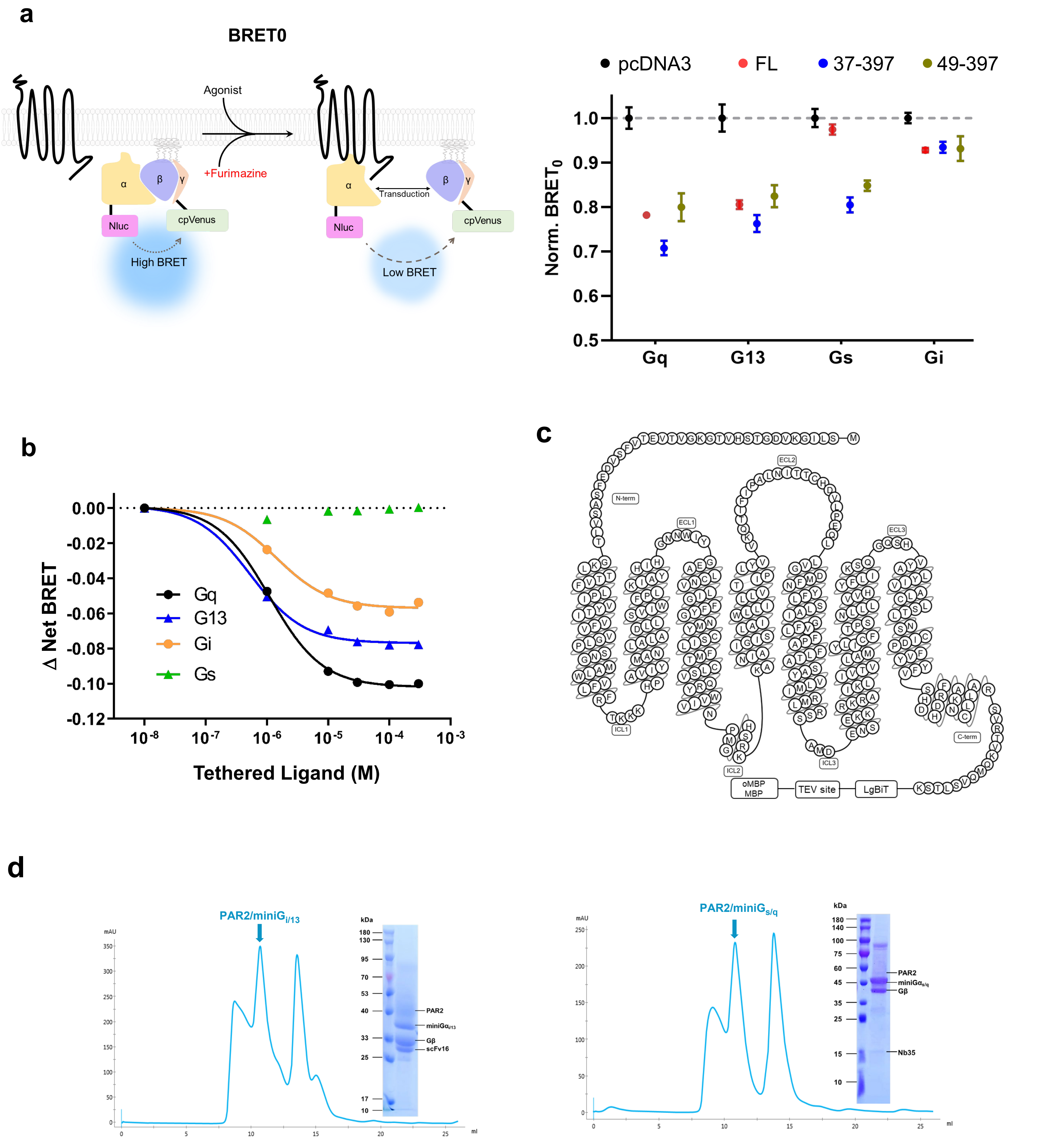

Supplementary Fig. S1: Expression and purification of PAR2. a A BRET0 assay to measure the intrinsic activity of PAR2 constructs. Left panel, a schematic diagram of the BRET0 assay; right panel, the intrinsic activity of PAR2 constructs in the BRET0 assay. Data are presented as mean values ± SD; n = 3 independent experimental replicates. **b** BRET activity of the tethered peptide to FL-PAR2. Data are presented as mean values ± SD; n = 3 independent experimental replicates. **c** A snake-shape diagram of the PAR2 construct used in complex assembling, the diagram was adopted from GPCRdb. **d** Size exclusion column profiles and SDS-PAGE analysis of PAR2/G-protein complexes.

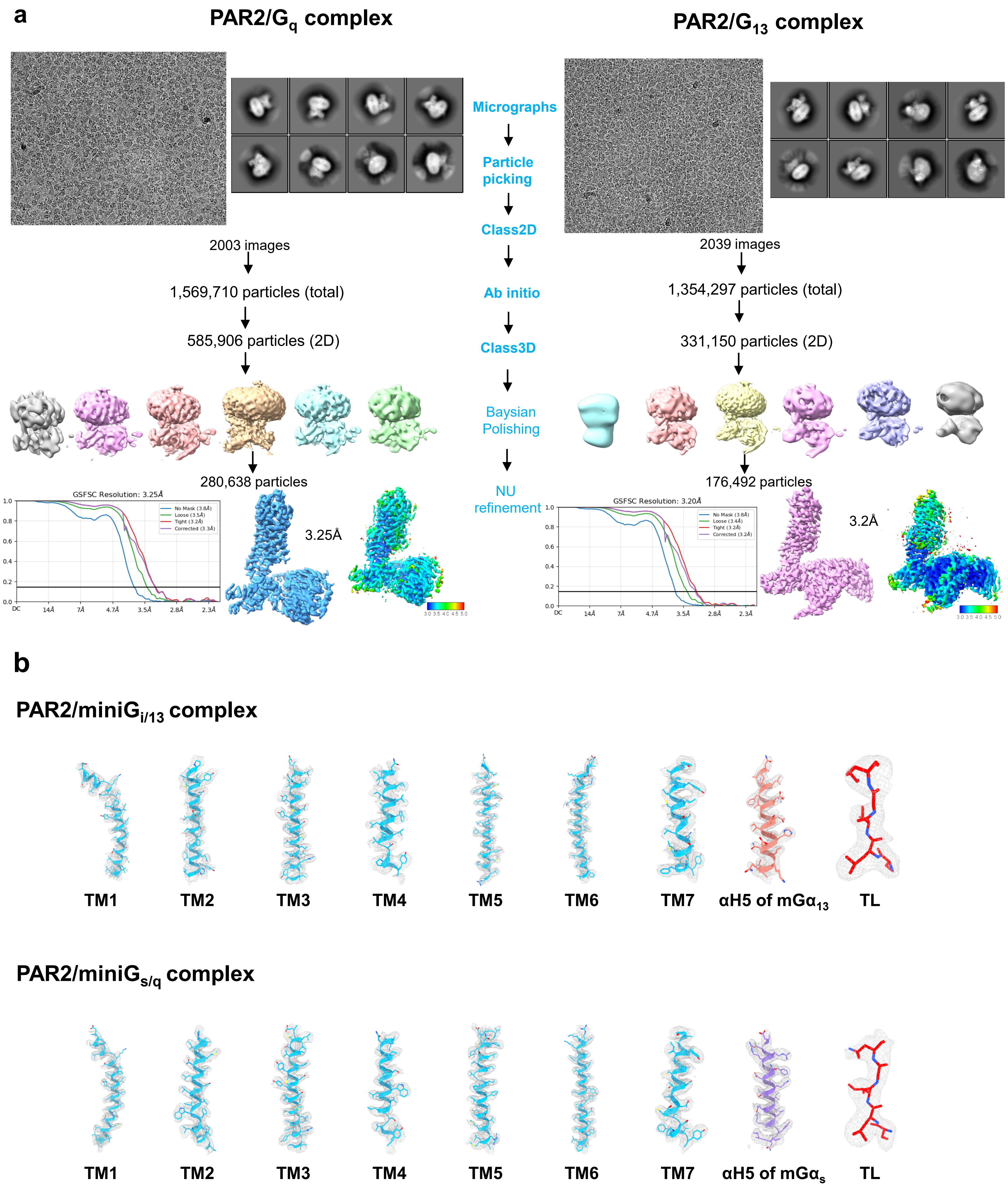

Supplementary Fig. S2: Single particle analysis of PAR2/G-protein complexes. a Flow-chart of cryo-EM data process of PAR2/G-protein complexes. **b** Cryo-EM density map of representative regions of PAR2/G-protein complexes.

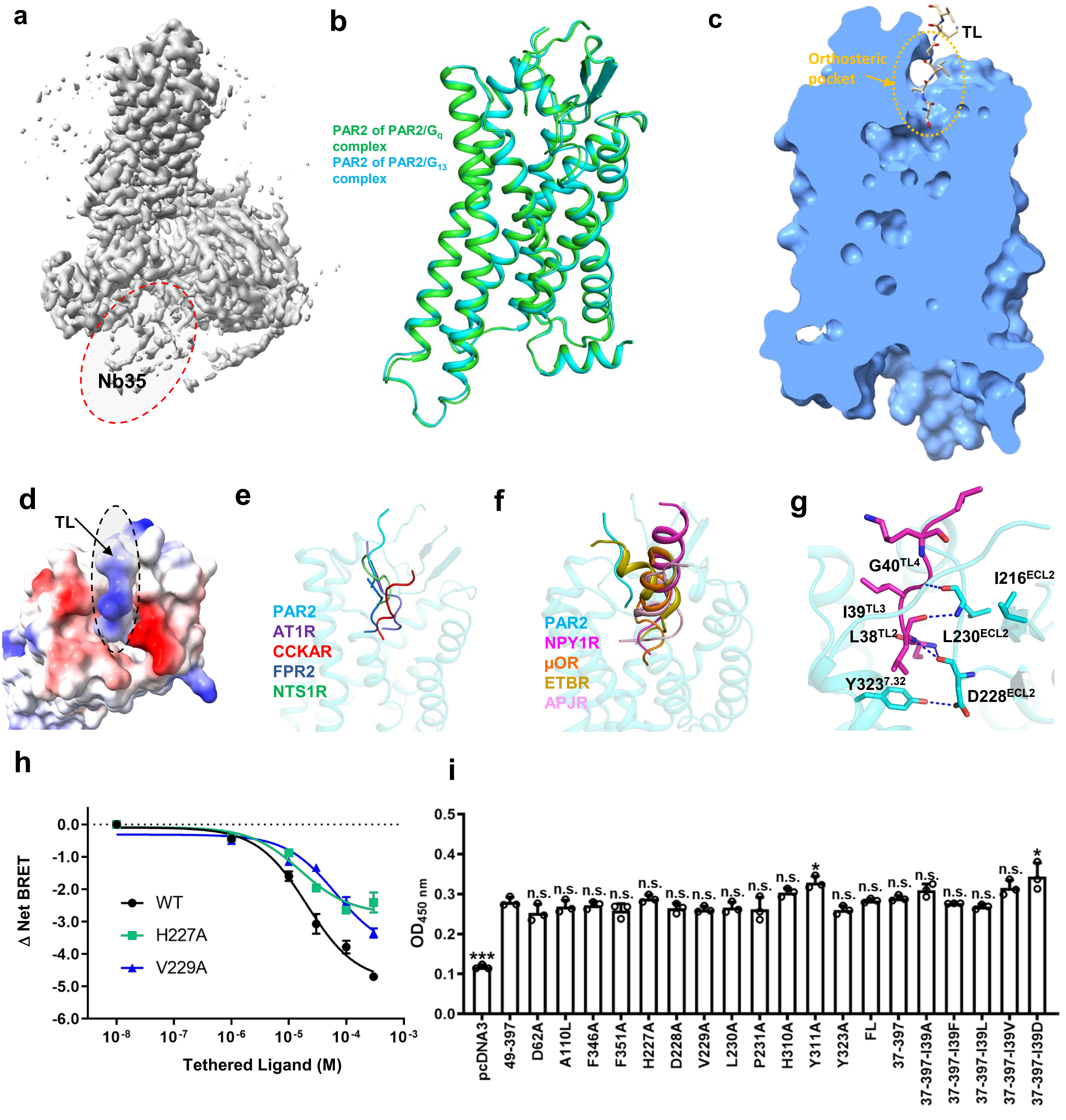

Supplementary Fig. S3: Additional information on the ligand binding pocket of PAR2. **a** Cryo-EM map of PAR2/G_q complex. The dashed circle indicates Nb35 region. **b** A comparison of the overall receptor conformation in G_q and G_{13} complexes. **c** A clipping view of the ligand binding pocket and the binding of TL to PAR2. **d** A surface electrostatic analysis of PAR2/G_{13} complex. **e-f** Comparison of peptide binding mode among peptide receptor family. NTSR1 (PDB:6os9), FPR2 (PDB:6omm), CCKAR (PDB:7ezh), AT1R (PDB:6os0), μOR (PDB:8f7x), NPY1R (PDB:7vgx), ETBR (PDB:8hbd), APJR (PDB:7w0p). **g** A detailed analysis of TL/PAR2 interactions, focusing on D228 and L230. **h** BRET2 G_{13} dissociation assay of PAR2 mutants. Data are presented as mean values ± SD; n = 3 independent experimental replicates. A statistical evaluation is listed in Supplementary Table S1. **i** Surface expression assay of PAR2 mutants. Data are presented as mean values ± SD; n = 3 independent experimental replicates. n.s., not significant; *, p<0.05; **, <0.01; ***, p<0.001; two-side *T*-test. Mutants for the TL dose-response analysis were generated based on the 49-397 PAR2 construct, which exhibits minimal intrinsic activity and serves as the wild-type receptor for statistical comparisons.

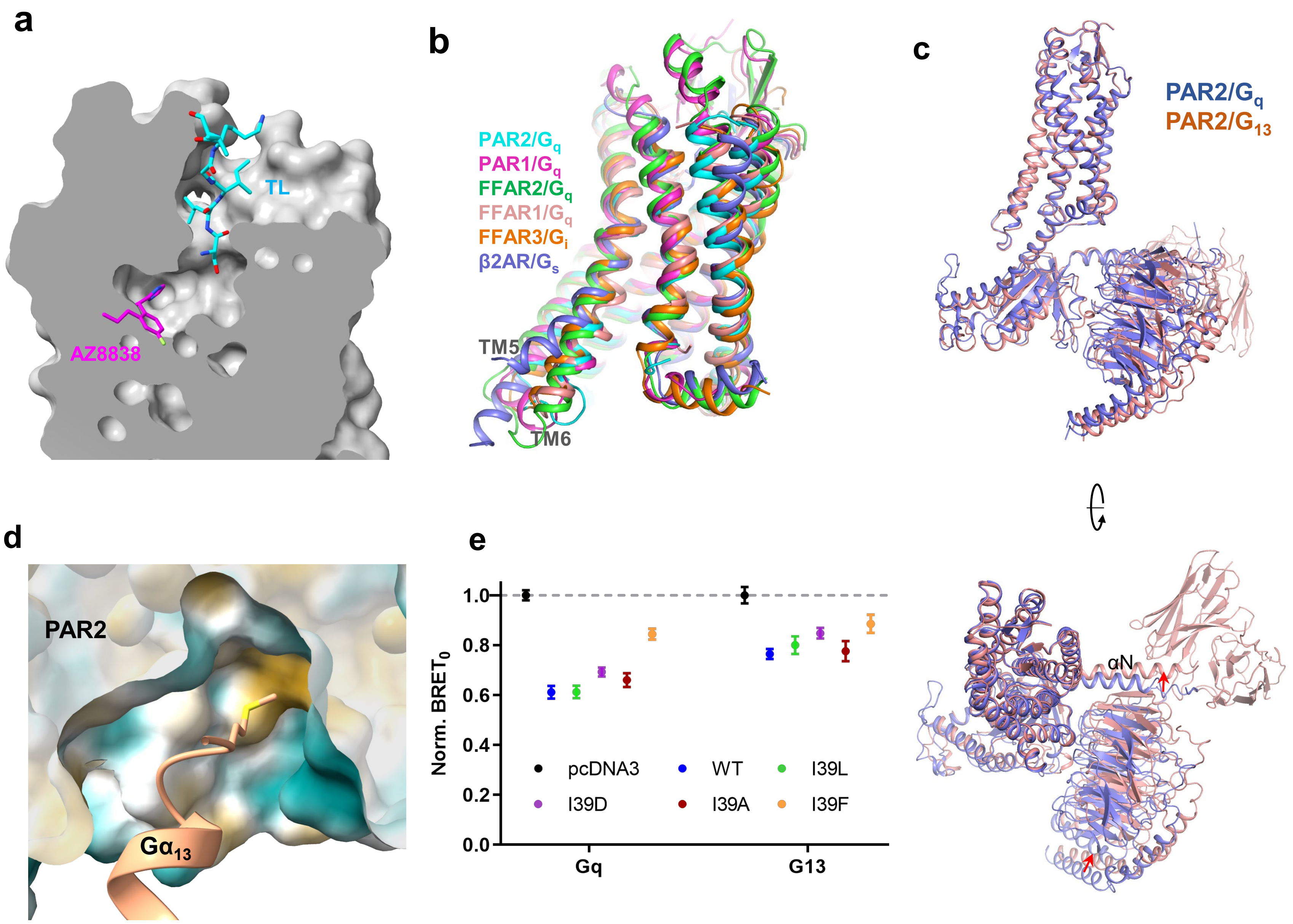

Supplementary Fig. S4: Additional information on receptor activation and G-protein coupling of PAR2. a A clipping view of ligand binding pocket superimposed by both TL and antagonist AZ8838 (PDB: 5ndd). **b** A structural comparison of PAR2 with related receptors. PAR1/G_q (PDB:8xor), FFAR2/G_q (PDB:8t3s), FFAR1/G_q (PDB:8eit) and FFAR3/G_i (PDB:8j21) and β2AR/G_s (PDB:3sn6). **c** A comparison of the overall G_q and G_{13} engagements of PAR2. **d** M228$^{G.H5.24}$ of Gα$_{13}$ inserts into a hydrophobic cavity of PAR2 on the intracellular side in the PAR2/G_{13} complex. **e** BRET0 assay for the intrinsic activity of Δ(1-36) truncation constructs of PAR2. Data are presented as mean values ± SD; n = 3 independent experimental replicates.

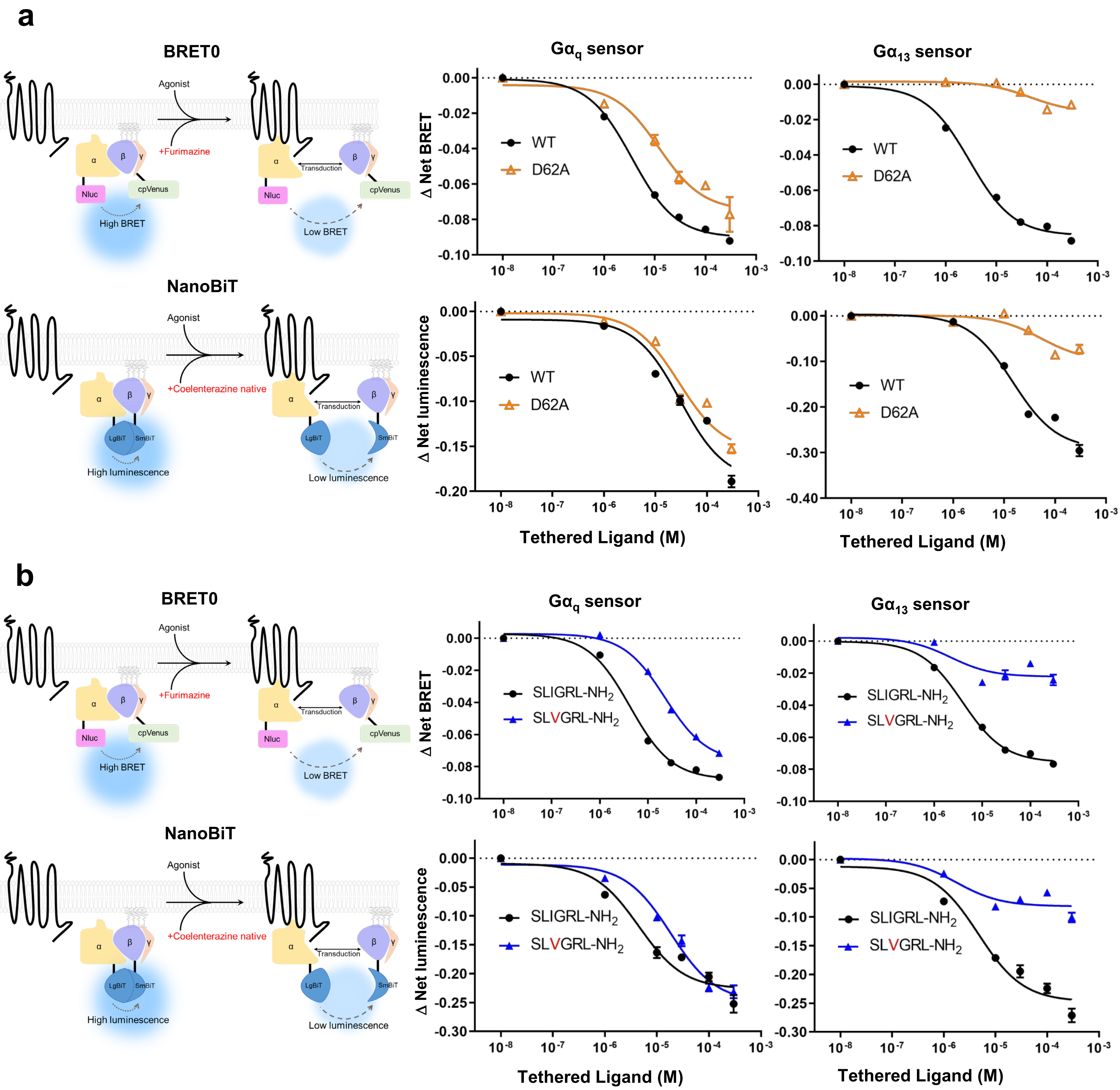

Supplementary Fig. S5: Additional data of biased signaling of PAR2. **a** Examination of G-protein coupling activity of PAR2 mutant via different assays. **b** Examination of G-protein coupling activity of the synthetic PAR2 AP peptides via different assays. Data are presented as mean values ± SD; n = 3 independent experimental replicates.

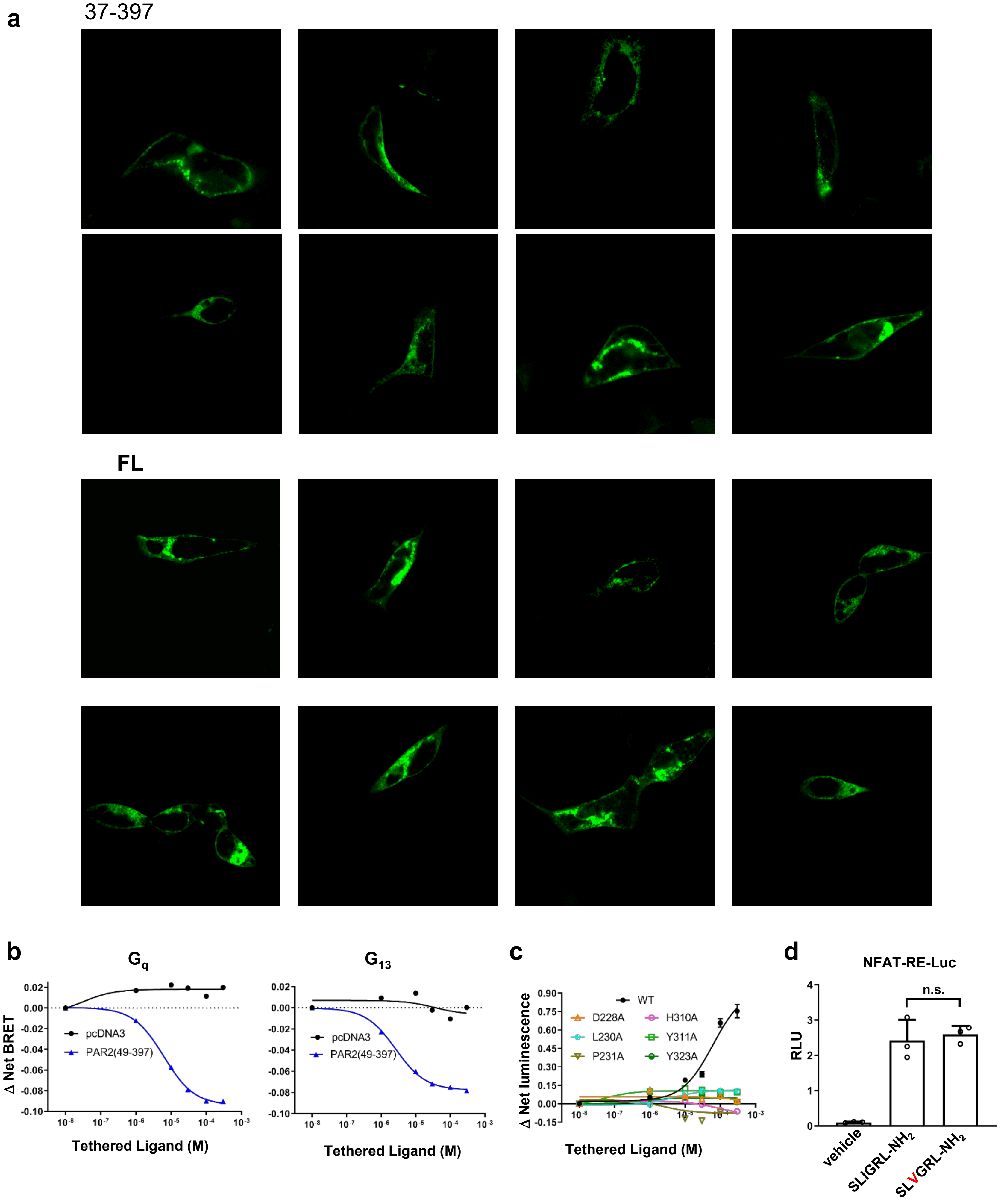

Supplementary Fig. S6: Additional functional assays of PAR2. **a** Representative confocal images of full-length and 37-397 constructs of PAR2. **b** A BRET assay of G_q and G_{13} on PAR2 construct and empty vehicle construct in AD293 cells. **c** A NanoBiT assay in which the LgBiT is fused to the C-terminus of PAR2 and SmBiT is fused to the C-terminus of Gβ in AD293 cells. Data are presented as mean values ± SD; n = 3 independent experimental replicates. **d** A NFAT-RE reporter assay of PAR2(49-397) construct treated by different tethered ligands. SLIGRL-NH_2 or SLVGRL-NH_2, 300 μM; data are presented as mean values ± SD; n = 3 independent experimental replicates. n.s., not significant; *$P < 0.05$; **$P < 0.01$; ***$P < 0.001$, two-side T-test. RLU, relative luciferase unit.

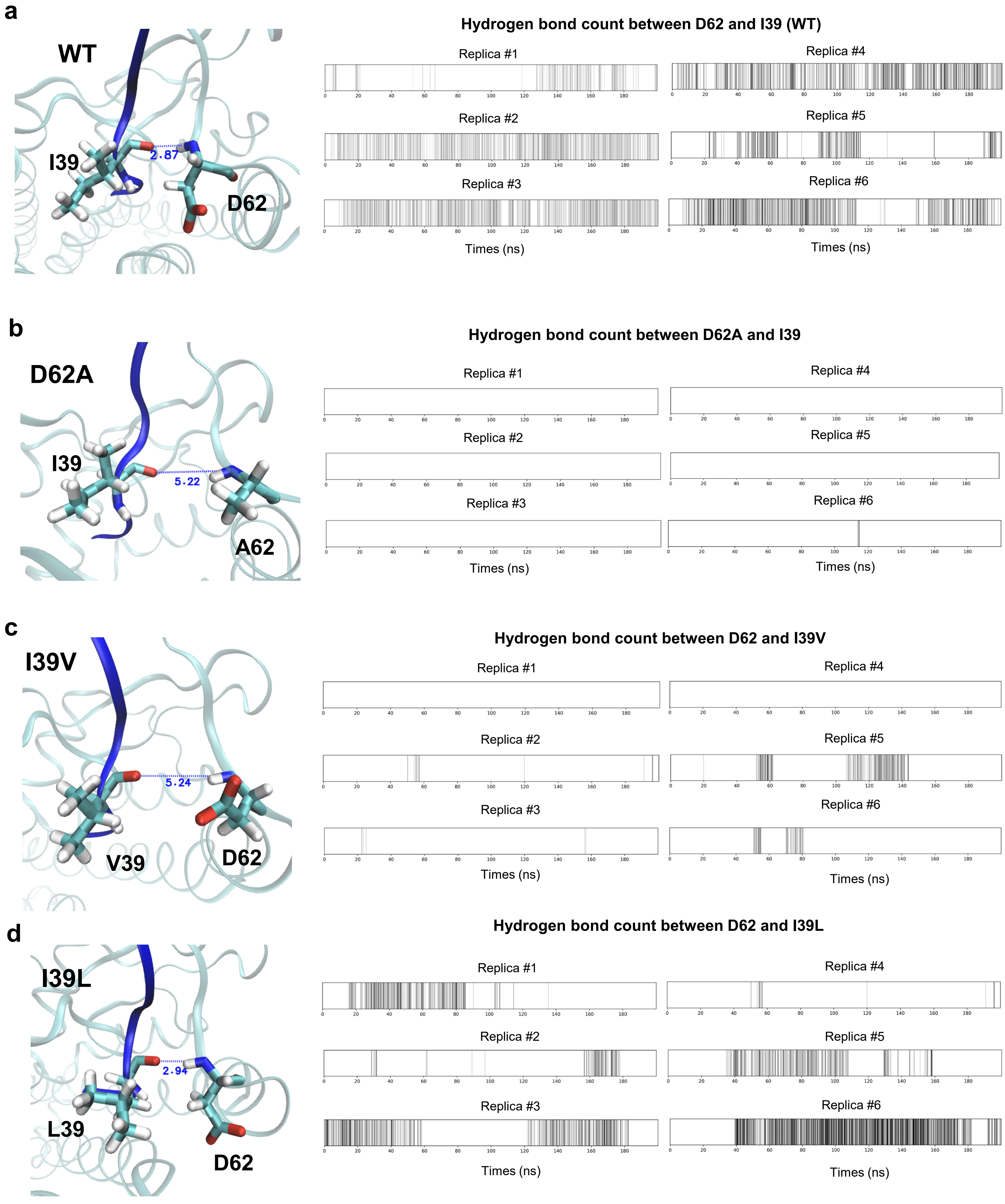

Supplementary Fig. S7: MD simulation analysis of PAR2. a-d MD simulations analysis the interaction between TL peptide and the N-terminal region of TM1 in WT (**a**), D62A (**b**), I39V (**c**) and I39L (**d**). Left panel, snap-shots of the I39(V, L)/D62(A) interactions; right panel, statistics of hydrogen bond interaction of I39(V, L)/D62(A) in MD simulations of PAR2, the cutoff of hydrogen bond is set to 3.0 Å with 20º tolerance.

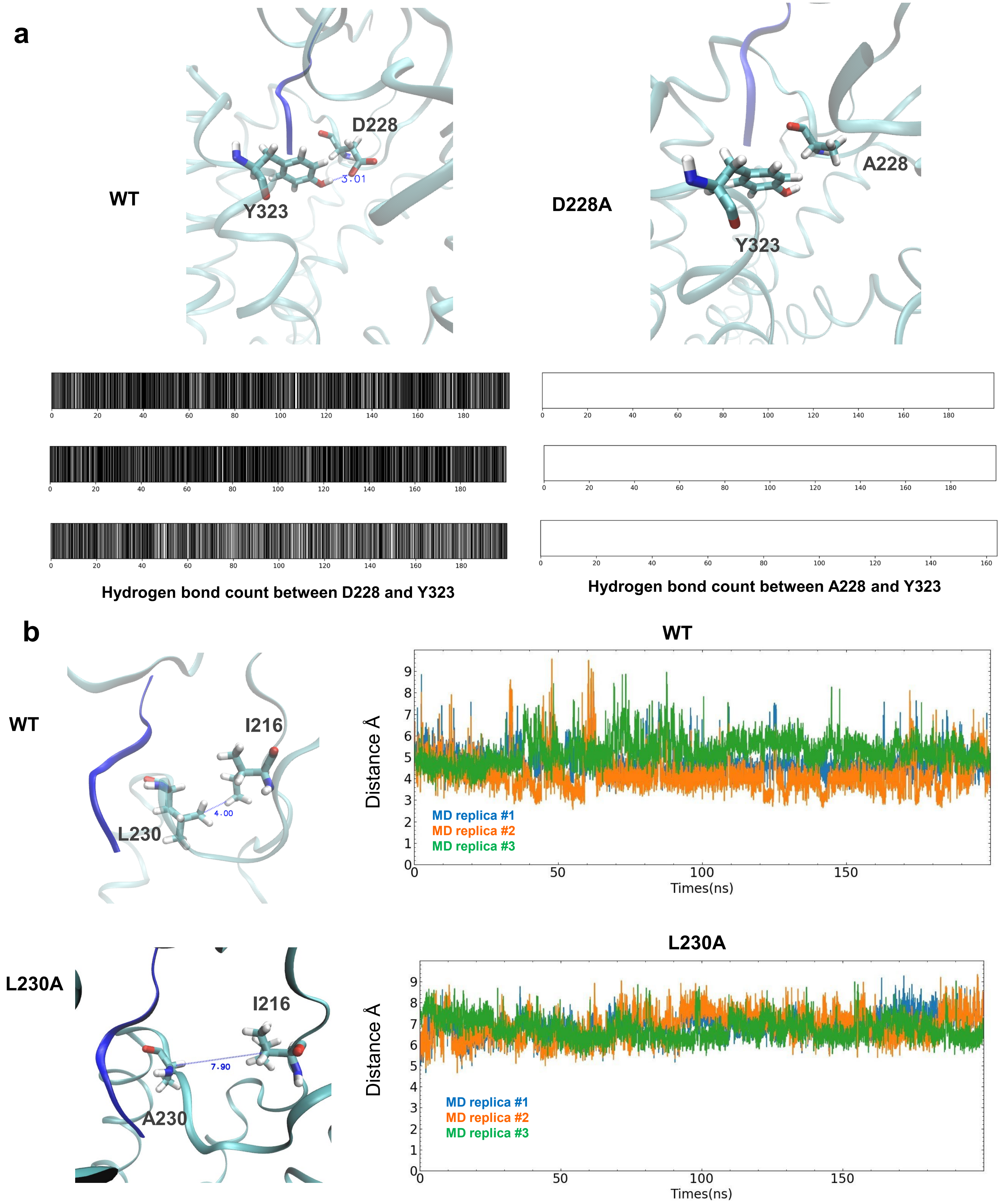

Supplementary Fig. S8: Additional MD simulation analysis of PAR2. **a** MD simulations analysis of the interaction between D228 and Y323 in PAR2. Hydrogen-bond interactions are shown as black bars along the timeline; absence of black bars indicates no hydrogen-bond formation. **b** MD simulation analysis of the inter-residue distance between L230 and I216 in PAR2. In the WT receptor, L230 maintains close contact with I216, whereas this distance is markedly increased in the L230A mutant.

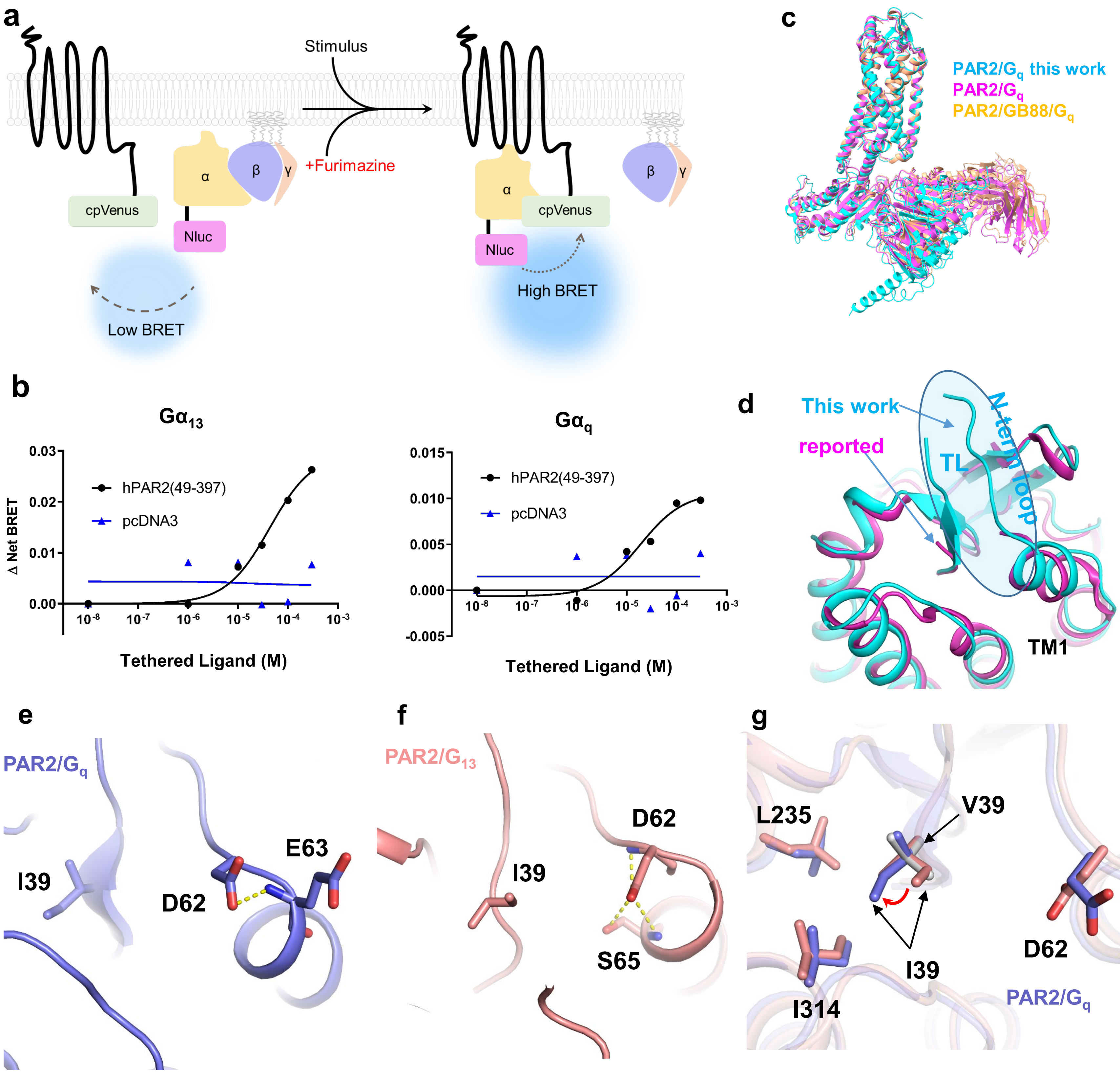

Supplementary Fig. S9: Additional data of PAR2 activation. **a** A schematic diagram of the BRET system. **b** The activity of added TL to the system. Data are presented as mean values ± SD; n = 3 independent experimental replicates. **c** A comparison of the overall PAR2 structure of this study with the reported study. PDB for the reported PAR2/G_q complex 9d0a (magenta), PAR2/GB88/G_q complex 9e7r (brown). **d** A detailed comparison of TL conformation of this study and reported one. **e** D62 forms hydrogen-bond with E63 in the PAR2/G_q complex. **f** D62 forms hydrogen-bonds with S62 in the PAR2/G_{13} complex. **g** I39 is able to form hydrophobic interaction with L235 and I314, but the ability is demolished when I39 changes to V39 due to the short side chain.

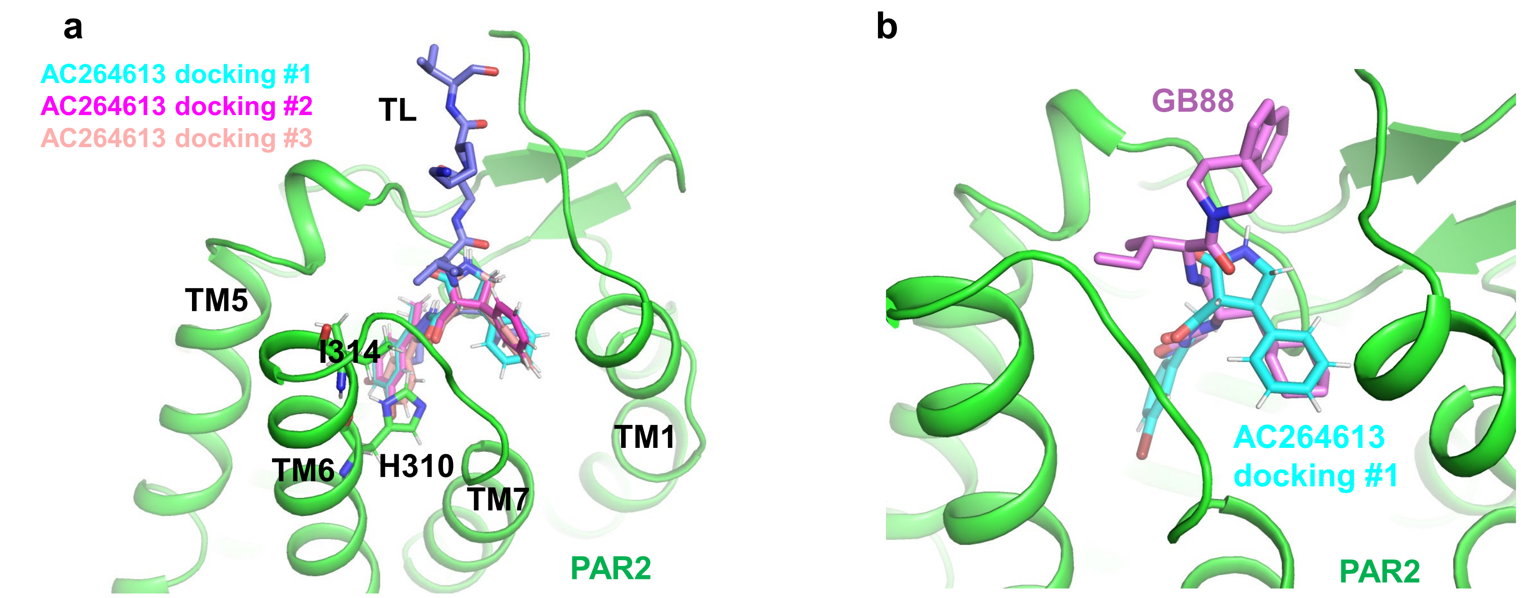

Supplementary Fig. S10: Molecular docking of small molecule agonist AC264613. a Top 3 docking pose of AC264313 in PAR2. **b** A comparison of the top 1 docking pose of AC264613 in PAR2 with the binding pose of GB88 to PAR2 (PDB:9e7r).

Supplementary Table S1: Pharmacologic characteristics of PAR2 ligand binding pocket mutants via BRET2 G_{13} dissociation assay.

	WT	D228A	L230A	P231A	H310A	Y311A	Y323A	H227A	V229A
EC_{50} (M)	2.050E-05	N.A.	N.A.	N.A.	N.A.	0.000006766, n.s.	N.A.	0.00001594, n.s.	0.00006507, n.s.
pEC_{50}	4.688	N.A.	N.A.	N.A.	N.A.	5.170	N.A.	4.797	4.187
SD(+/-)	0.0562	N.A.	N.A.	N.A.	N.A.	0.0913	N.A.	0.1092	0.1086
E_{max}	-4.756	N.A.	N.A.	N.A.	N.A.	-1.593, ***	N.A.	-2.682, **	-3.613, *
SD(+/-)	0.1539	N.A.	N.A.	N.A.	N.A.	0.0713	N.A.	0.1647	0.2679
n	3	3	3	3	3	3	3	3	3

Note: N.A., not available due to poor curve fit; n.s., not significant; *, p<0.05; **, p<0.01; ***, p<0.001; two-side T-test.

Supplementary Table S2: Pharmacologic characteristics of PAR2 biased mutants via BRET2 G_{13} and G_q dissociation assay.

$G\alpha_{13}$ sensor	WT	A110L	F346A	F351A	D62A
EC_{50} (M)	1.590E-05	0.00001281, n.s.	0.0000008533, **	N.A.	N.A.
pEC_{50}	4.799	4.892	6.069	N.A.	N.A.
SD(+/-)	0.0769	0.1012	0.2322	N.A.	N.A.
E_{max}	-1.942	-1.969, n.s.	-0.8974, **	N.A.	N.A.
SD(+/-)	0.0839	0.1092	0.1148	N.A.	N.A.
n	3	3	3	3	3

$G\alpha_q$ sensor	WT	A110L	F346A	F351A	D62A
EC_{50} (M)	6.454E-06	N.A.	0.00001051, *	0.00001415, *	0.00001452, *
pEC_{50}	5.190	N.A.	4.978	4.849	4.838
SD(+/-)	0.1423	N.A.	0.0751	0.1496	0.1701
E_{max}	-1.142	N.A.	-0.9699, n.s.	-0.809, n.s.	-1.217, n.s.
SD(+/-)	0.0790	N.A.	0.0388	0.0671	0.1153
n	3	3	3	3	3

Note: N.A., not available due to poor curve fit; n.s., not significant; *, p<0.05; **, p<0.01; ***, p<0.001; two-side T-test.

Supplementary Table S3: Pharmacologic characteristics of synthetic PAR2 peptides via BRET2 G_{13} and G_q dissociation assay.

$G\alpha_{13}$ sensor	SLIGRL-NH2	SLVGRL-NH2
EC_{50} (M)	6.753E-06	N.A.
pEC_{50}	5.171	N.A.
SD(+/-)	0.257	N.A.
E_{max}	-2.222	N.A.
SD(+/-)	0.2800	N.A.
n	3	3

$G\alpha_q$ sensor	SLIGRL-NH2	SLVGRL-NH2
EC_{50} (M)	1.145E-05	0.00001212, n.s.
pEC_{50}	4.941	4.917
SD(+/-)	0.0875	0.1557
E_{max}	-1.680	-1.268, n.s.
SD(+/-)	0.0794	0.1075
n	3	3

Note: N.A., not available due to poor curve fit; n.s., not significant; *, p<0.05; **, p<0.01; ***, p<0.001; two-side T-test.

Supplementary Video S1: Movie track of MD simulation trajectory of Δ(1-36) PAR2 (WT).
The length is 200ns, interval is 5 steps. The blue dash line and number indicate the distance between I39 and D62.

Supplementary Video S2: Movie track of MD simulation trajectory of Δ(1-36) PAR2 (D62A).
The length is 200ns, interval is 5 steps. The blue dash line and number indicate the distance between I39 and A62.

Supplementary Video S3: Movie track of MD simulation trajectory of Δ(1-36) PAR2 (I39V).
The length is 200ns, interval is 5 steps. The blue dash line and number indicate the distance between V39 and D62.

Supplementary Video S4: Movie track of MD simulation trajectory of Δ(1-36) PAR2 (I39L).
The length is 200ns, interval is 5 steps. The blue dash line and number indicate the distance between L39 and D62.
